# Non-Temperature-Induced Antitumor Effects of Amplitude-Modulated Radiofrequency: Molecular and Functional Synergies with Radiotherapy

**DOI:** 10.3390/cancers18101613

**Published:** 2026-05-16

**Authors:** Paraskevi Danai Veltsista, Wolfgang Walther, Sebastian Torke, Andranik Ivanov, Anna Dieper, Dieter Beule, Daniel Zips, Ulrike Stein, Pirus Ghadjar

**Affiliations:** 1 Department of Radiation Oncology, Charité—Universitätsmedizin Berlin, 13353 Berlin, Germany; anna.dieper@charite.de (A.D.); daniel.zips@charite.de (D.Z.); pirus.ghadjar@charite.de (P.G.); 2 Experimental and Clinical Research Center, Charité—Universitätsmedizin Berlin and Max-Delbrück-Center for Molecular Medicine, 13125 Berlin, Germany; wowalt@mdc-berlin.de (W.W.); sebastian.torke@mdc-berlin.de (S.T.); ustein@mdc-berlin.de (U.S.); 3 German Cancer Consortium (DKTK), 69120 Heidelberg, Germany; 4 Core Unit Bioinformatics, Berlin Institute of Health, Charité—Universitätsmedizin Berlin, 10117 Berlin, Germany; andranik.ivanov@bih-charite.de (A.I.); dieter.beule@bih-charite.de (D.B.)

**Keywords:** hyperthermia, hyperthermic oncology, non-temperature-induced effects, RF, AMRF, EMF

## Abstract

Background/Objectives: Amplitude-modulated radiofrequency (AMRF) fields have emerged as promising non-temperature-induced strategies in oncology. While conventional hyperthermia (HT) relies on thermal stress, the biological impact of AMRF, particularly in combination with radiotherapy (RT), remains insufficiently characterized. Methods: We assessed RF and AMRF, alone or with RT, using phenotypic analyses of proliferation, apoptosis, and necrosis across four cancer cell lines (HT29, SW620, U343, U138). Transcriptomic profiling with Kyoto Encyclopedia of Genes and Genomes (KEGG), GO:BP, and Reactome enrichment was performed in SW620 and U138 cells, selected for their strong phenotypic responses. Results: Across the panel, AMRF was associated with broader cytotoxic responses than RF or HT in most but not all cell lines. AMRF+RT produced the strongest necrotic responses, with cell-line-specific exceptions identified explicitly in the Results (the absence of a significant AMRF+RT apoptotic effect in SW620 and the absence of a significant AMRF+RT necrotic response in U343). In SW620 cells, AMRF was associated with extensive transcriptional reprogramming involving immune modulation, extracellular matrix remodeling, and cell cycle regulation, whereas RF alone showed narrower and delayed effects. In contrast, U138 cells showed elevated apoptosis and necrosis but limited transcriptional changes—a phenotype–transcriptome divergence that points to mechanisms operating downstream of transcription and warrants functional investigation in dedicated follow-up studies. Conclusions: AMRF and AMRF+RT emerge as promising non-temperature-induced anticancer modalities in the cell-line models profiled here, with the pattern of response varying between cell lines. These findings expand the biological impact of RF-based treatments and set the grounds for further investigation in mechanistic and translational studies.

## Simple Summary

Non-temperature-induced effects of radiofrequency electromagnetic fields represent an emerging area in oncological research, distinct from conventional hyperthermia which relies on thermal cytotoxicity. This study aimed to systematically characterize the biological impact of amplitude-modulated radiofrequency (AMRF), alone and in combination with radiotherapy, using colorectal cancer and glioblastoma cell models. Through integrated phenotypic and transcriptomic analyses, we show that AMRF was associated with broader cytotoxic and transcriptional responses than unmodulated radiofrequency or heat alone in most cell lines profiled, with AMRF combined with radiotherapy producing the strongest necrotic responses (subject to cell-line-specific exceptions reported in the Results). Colorectal and glioblastoma cells exhibited divergent response patterns, consistent with tumor-type-specific differences in treatment sensitivity. These findings establish AMRF as a biologically distinct, non-temperature-induced anticancer modality and provide a molecular foundation for its further translational investigation as an adjunct to radiotherapy.

## 1. Introduction

The biological effects of radiofrequency electromagnetic fields (EMFs) have been a subject of controversy for decades. While EMFs can be applied as continuous radiofrequency (RF) waves or modified by amplitude modulation (AMRF), their impact extends beyond conventional hyperthermia (HT)-induced cytotoxicity. In the context of oncological research, non-temperature-induced effects are gaining increasing attention, distinguishing energy deposition mechanisms that do not lead to measurable temperature increases from classical hyperthermic effects [1,2]. These effects are often referred to as “nonthermal” in the literature, though this terminology remains imprecise. Instead, non-temperature-induced effects more accurately describe energy absorption mechanisms, including isothermal energy deposition, wherein a change in internal energy occurs without an associated temperature rise due to efficient heat dissipation or slow energy transfer [3].

A non-temperature-induced biological effect can be considered valid if exposure to RF or AMRF elicits a measurable physical or biological response under identical conditions, with precise temperature control in the treated sample. However, the characteristics of these effects may vary as a function of temperature, and identifying the biophysical mechanisms involved remains a key challenge. Over the past decades, RF has been widely used in oncology under the framework of HT therapy, primarily in combination with chemotherapy or radiotherapy (RT) [4]. However, conventional HT applications require precise temperature elevation beyond 41 °C to achieve significant therapeutic effects, limiting their feasibility for broader oncological applications due to technical and physiological constraints [5,6,7].

Recent preclinical studies have provided substantial evidence that RF and AMRF induce biological effects independent of temperature, suggesting a novel avenue for their application in cancer research. Several in vitro and in vivo investigations have demonstrated non-temperature-induced effects of RF at 13.56 MHz, with capacitive coupling delivering energy to tumor cells at controlled sub-hyperthermic conditions [8,9]. The role of amplitude modulation in enhancing these effects remains an open question, as both RF and AMRF have been associated with distinct antitumor properties when compared with conventional water bath heating at 42 °C [3,9]. However, the precise molecular and biophysical mechanisms underlying these effects remain poorly understood.

Experimental evidence suggests that electromagnetic fields may exert biological effects through non-temperature-dependent mechanisms such as membrane potential modulation, ion channel activation, and cellular signaling pathway alterations. Calcium flux regulation has been identified as a potential mediator of AMRF-induced effects at 147 MHz, particularly at low modulation frequencies (e.g., 16 Hz), highlighting the need to further investigate cellular electrophysiology under RF exposure. In addition to membrane perturbations, non-temperature-induced effects have been proposed to influence gene expression, intracellular oxidative stress, and immune modulation, offering a potential rationale for their observed antitumor effects [2,10,11]. Building on these non-temperature-induced mechanisms, early clinical use of biofeedback-derived tumor-specific frequencies was feasible and well tolerated, with signals of antitumor activity in advanced cancers, including objective responses and durable disease stabilization [11,12,13].

Despite growing experimental support, the reproducibility and quantification of non-temperature-induced effects remain a major challenge. A key limitation has been the absence of standardized methods for distinguishing true RF- and AMRF-induced biological responses from hidden thermal effects, including localized heating and cellular microenvironment changes. The lack of direct comparative studies between water-bath HT and RF/AMRF exposure has also kept the biophysical validity of non-temperature-induced mechanisms a matter of debate. A controlled experimental framework that systematically excludes thermal confounders is therefore needed before the field can advance [14].

In the present study, we aimed to systematically investigate the non-temperature-induced effects of RF and AMRF by employing a controlled in vitro approach using colorectal cancer—CRC (SW620, HT29) and glioblastoma—GBM (U343, U138) cell lines. CRC was selected due to its global prevalence and therapeutic challenges. Despite being the third most common cancer worldwide, treatment options remain limited—particularly for patients with metastatic disease, who face poor long-term survival [15,16]. In parallel, GBM was chosen as a model of high-grade glioma where standard radiotherapy is limited by intrinsic cellular radioresistance, tumor hypoxia, and DNA-repair capacity [17,18], while infiltrating cancer cells and a refractory tumor microenvironment further constrain treatment outcomes [19]. Modulated electrohyperthermia has shown clinical signals in retrospective cohorts of relapsed glioma and astrocytoma [20].

To capture disease heterogeneity and enhance translational relevance, we selected SW620, a metastatic CRC model derived from lymph node metastasis [21,22,23], and HT29, a primary tumor-derived line [24,25]. This allowed us to assess RF/AMRF responses across different stages of CRC progression. Likewise, U343 and U138 glioblastoma cell lines, which differ in genetic background and phenotypic profiles, were chosen to reflect the diversity of GBM biology [26,27,28,29]. These well-characterized models are widely used in preclinical oncology research and offer the experimental reliability necessary for mechanistic investigation. Our previous work established preliminary evidence of non-temperature-induced anticancer effects in CRC models [3]. However, the impact of these mechanisms in other cancer types, such as GBM, remains unexplored. By including both tumor types, we aimed to assess the context-dependency and translational relevance of AMRF-based therapeutic interventions.

Based on initial phenotypic responses, RNA-sequencing was subsequently focused on SW620 and U138, which exhibited the most promising differential effects upon treatment.

## 2. Materials and Methods

### 2.1. Experimental Setup

RF and AMRF treatments were conducted using the LabEHY-200 system (Oncotherm Kft., Budapest, Hungary), a device optimized for preclinical in vitro studies involving cell suspensions. The system operates at a fixed carrier frequency of 13.56 MHz and allows optional amplitude modulation (AM). For AMRF exposures, a band-limited 1/f noise (pink noise) with a lower corner frequency $f_{0}=100$ Hz was used at modulation index m=50%. Target temperature was 42 °C [3]. The upper cutoff of the AM spectrum is set by the analog circuitry of the LabEHY-200 RF generator and is not exposed at the controller-software level. The manufacturer characterizes the AM band as audio-frequency [3].

**Physical dosimetry.** The LabEHY-200 system was operated at a nominal adjusted power $P_{\mathrm{adj}}=10$ W, with reflected power typically <5% [3]. The full dosimetric characterization of the sandwich in vitro applicator used in the present study is reported in Ref. [3]. In cancer-cell suspensions (Ref. [3], Figure 3A), the corrected temperature gradient ${\left(\Delta T/\Delta t\right)}_{\mathrm{corr}}$ is systematically about 15–20% higher under RF than under AMRF, corresponding via $\mathrm{SAR}\;\left[W/\mathrm{kg}\right]=60\times {\left(\Delta T/\Delta t\right)}_{\mathrm{corr}}\;\left[{}^{\circ}C/\min\right]$ (Equation (1) of Ref. [3]) to a thermal SAR in the range 200–240 W/kg. Ref [3] (Section 3.1, p. 11) interprets this difference as evidence of isothermal energy storage under amplitude modulation. The corresponding driving electric-field magnitude at the sample is given by $E\;\left[V/m\right]=45\times {\left(\mathrm{SAR}\;\left[W/\mathrm{kg}\right]/\sigma \;\left[S/m\right]\right)}^{1/2}$ (Equation (1) of Ref. [9]) with conductivity σ=1.2 S/m for RPMI medium. The in vitro applicator comprised grid-shaped steel electrodes (4×1.6×0.75 cm^2^) separated by 1.3 cm, with cell suspensions enclosed in a sealed chamber between coverslips, as described in Ref. [3]. The corresponding clinical AMRF range at 13.56 MHz is 1–20 W/kg/45–200 V/m [3]. The in vitro values exceed this range because of the geometric efficiency of direct capacitive coupling in the in vitro chamber, but biological interpretations in this study rest on matched-temperature water-bath controls, which control for any absolute-SAR differences between the in vitro and clinical regimes. No new physical measurements were performed for the present manuscript. The dosimetric parameters above are taken from Refs [3,9], which provide the complete physical characterization of this apparatus.

The in vitro applicator was designed to provide uniform exposure and thermal stability. It features a symmetric sandwich-like architecture on both the upper and lower sides of the treatment chamber. Each side consists of a plastic frame, glass cover, grid-shaped electrode, water flow layer in direct contact with the electrode, and a coverslip. The cell suspension (1.5 mL) is enclosed between two such coverslips, forming a sealed chamber with consistent thermal contact, while the closed-loop water-circulation system enforces precise thermal regulation during exposures. This geometry precludes any direct galvanic contact between the electrodes and the cell suspension. Temperature in the cell chamber was directly monitored throughout each exposure with a fluoroptic sensor (FLUOROPTIC^®^ THERMOMETER m3300, Luxtron, Advanced Energy Industries, Inc., Denver, CO, USA, ⌀=0.5 mm) positioned at the chamber centre (Ref. [3], Section 2.3). As a quantitative bound on hidden local hot-spots, Ref. [9] (p. 2) and Ref. [3] (Equation (3)) establish that a local SAR of 10,000 W/kg focused into a 1 mm radius spherical volume would produce a maximal temperature increase of only 2 °C.

A schematic overview of the LabEHY-200 system and in vitro applicator is provided in our previously published work [3] and illustrates the technical configuration used in this study.

### 2.2. Experimental Modes

To investigate the biological effects of radiofrequency-based therapies, four distinct treatment settings were applied, each involving 1.5 mL of single-cell suspension ($1\times 10^{6}$ cells/mL) placed in a sealed chamber. Treatments were conducted at 42 °C for 65 min, with the temperature controlled via an external water flow to ensure isothermal conditions.

In the RF condition, cells were exposed to a continuous radiofrequency field at 13.56 MHz without modulation. For AMRF treatment, the same carrier frequency was amplitude-modulated as described above. To differentiate thermal from non-temperature-induced effects, water bath (WB) controls were included at 37 °C, 42 °C, and 44 °C. The 37 °C WB served as the negative control, while the 44 °C WB acted as a positive control for heat-induced cytotoxicity.

To assess the combinational effects of RF-based treatment and ionizing radiation, an additional set of conditions was included in which cells were irradiated with 1×8 Gy γ-radiation immediately prior to RF or AMRF exposure. For these combination treatments (RF+RT and AMRF+RT), additional controls included irradiated and non-irradiated WB samples at both 37 °C and 42 °C. The 37 °C + RT control specifically accounted for the effects of radiation alone. This design enabled systematic dissection of temperature-, radiation-, and EM field-dependent biological effects.

### 2.3. Cell Lines and Culture

Cells used in this study were obtained from the American Type Culture Collection (ATCC, Manassas, VA, USA) or the German Collection of Microorganisms and Cell Cultures GmbH (Leibnitz Institute DSMZ, Braunschweig, Germany) (Table 1). Cell cultures were maintained in either DMEM or RPMI 1640 medium (Thermo Fisher Scientific, Waltham, MA, USA) supplemented with 10% fetal bovine serum (FBS) (Bio & Sell, Feucht, Germany) and 1% Penicillin/Streptomycin 100× (Capricorn Scientific, Ebsdorfergrund, Germany). All cells were incubated at 37 °C in a humidified atmosphere containing 5% CO_2_. Cells were used for experiments upon reaching a confluency of 70%. Subculture ratios and seeding densities were varied across the parallel maintenance flasks according to the planned experimental window for the week, as described below. All cell lines were maintained at low passage and used for experiments within the following passage-number caps: passage ≤15 for the colorectal cancer lines (HT29, SW620) and passage ≤12 for the glioblastoma lines (U138, U343). To avoid the cumulative phenotypic drift that would result from repeated re-splitting of a single flask through high passage, several T75 flasks (75 cm^2^ growth area) of each cell line were maintained in parallel and synchronized to a fixed three-experiment-per-week schedule. On each experiment day, one flask was used for the experiment, while the remaining flasks were re-split to deliver the appropriate confluency on the following experiment day: 1:6 for use 2 days later, or 1:12 with a mid-cycle medium change for use 4 days later. This staggered schedule kept every experimental flask at the appropriate density (70% confluency) on the day it was needed, without prior over-passaging. All cell lines were tested for mycoplasma contamination weekly using the MycoAlert—Mycoplasma detection kit (Lonza, Basel, Switzerland). Only mycoplasma-negative cultures were used for any RF, AMRF, or matched water-bath control exposure. Plastic consumables for cell maintenance and experiments were used from BD Biosciences (Heidelberg, Germany), Sartorius (Göttingen, Germany), or Sarstedt (Nümbrecht, Germany).

All exposures (RF, AMRF, combination with RT, and the matched water-bath temperature controls at 37 °C, 42 °C, and 44 °C) were conducted in the same sealed chamber or matched sealed tubes, using the same RPMI-1640 or DMEM medium supplemented with 10% FBS and 1% Penicillin/Streptomycin, for the same 65 min exposure duration. Any drift in chamber chemistry (pH, osmolality or dissolved gases) intrinsic to this chamber geometry and medium chemistry therefore contributes equally to the treatment and the matched-temperature water-bath control arms, and cannot account for any treatment-specific effect reported below. The standard bicarbonate buffering of RPMI-1640 (2.0 g/L NaHCO_3_) and DMEM (3.7 g/L NaHCO_3_) is adequate to maintain physiological pH over this time-frame under limited CO_2_ egress from a sealed chamber.

### 2.4. Treatment and Cell Populations

The attached cells were washed with PBS and trypsin (Gibco™, Carlsbad, CA, USA) was applied to detach the cells and create a cell suspension. The cells were then counted using Countess™ (Invitrogen, Waltham, MA, USA) and EVE™ cell counting chambers (Science Services GmbH, Munich, Germany). The cell suspension was then adjusted to a final concentration of $1\times 10^{6}$ cells/mL. Immediately prior to loading into the sealed in vitro chamber, each suspension was briefly homogenized by mild vortexing (low speed, a few seconds) to disperse any sedimentation-induced density gradients. For water-bath controls, the 1.5 mL cell suspension was placed in a 15 mL screw-cap polypropylene tube (Sarstedt, Nümbrecht, Germany. 17 mm × 120 mm) and incubated in Precision GP 02™ water baths (Thermo Scientific, Waltham, MA, USA) at the matched control temperature (37 °C, 42 °C, or 44 °C). The fluoroptic temperature sensor (FLUOROPTIC^®^ THERMOMETER m3300, Luxtron, Section 2.1) was inserted through the screw cap to monitor in-suspension temperature throughout the exposure. For irradiation (RT, HT+RT, RF+RT, and AMRF+RT arms), the Biobeam GM 2000 (Gamma-Service Medical GmbH, Leipzig, Germany) was used. For combination treatments (RF+RT and AMRF+RT), the cell suspension was exposed to 1×8 Gy γ-radiation in the Biobeam GM 2000 and then transported to the LabEHY-200 chamber. The mean elapsed time from completion of γ-irradiation to onset of RF/AMRF exposure was 10–15 min.

### 2.5. Cell Viability and Proliferation

Cell proliferation was monitored using the IncuCyte^®^ ZOOM System (Essen BioScience, Ann Arbor, MI, USA). Directly after the experiment, 30 µL of cell suspension was spun down and re-diluted in 300 µL of YOYO™-3 Iodide (Invitrogen, Waltham, MA, USA—Y3606—1 mM Solution in DMSO) in a final concentration of 250 nM. Subsequently, every mixture was used in technical triplicates of 100 µL each. In Sarstedt (Nümbrecht, Germany) 96-well plates, $10^{4}$ cells/well (10,000 cells/well) were seeded. In every well, we applied 100 µL of the cell/YOYO3 mix and we added 3 µL of Annexin V Recombinant Protein—FITC (Thermo Fisher Scientific, Waltham, MA, USA, Cat. No. BMS306FI-100). The plates were incubated and monitored for 4 days (96 h) at 37 °C in a humidified incubator with 5% CO_2_. The analysis of cell viability and proliferation was performed using the integrated software IncuCyte ZOOM software 2016B (Essen BioScience, Ann Arbor, MI, USA). Pre-defined segmentation masks and fluorescence thresholds for Annexin V (apoptosis) and YOYO-3 (necrosis) were applied uniformly to every well. No per-condition parameter adjustment or selective re-analysis was performed. Formal analyst-level blinding to treatment condition was not implemented in the present study (see Section 4, Limitations).

### 2.6. RNA Extraction

After seeding part of the cells for cell death and proliferation analysis, the rest of the cell suspensions were divided in half. One part was placed in a 10 cm petri dish (Sigma-Aldrich, Burlington, MA, USA) and incubated for 24 h at 37 °C in a humidified incubator with 5% CO_2_. The other part was placed in a 1.5 mL Eppendorf tube (Eppendorf, Hamburg, Germany), centrifuged for 5 min (1200 RPM, room temperature), washed with PBS, centrifuged again, and stored at −80 °C. Total RNA was isolated from both parts of every sample, using the RNA extraction kit from Roboklon (Roboklon, Berlin, Germany) according to the instructions of the manufacturer. The RNA was quantified with NanoDrop™ 2000/2000c Spectrophotometers (Peqlab, Erlangen, Germany).

### 2.7. RNA Sequencing

For RNA sequencing, at least three independent biological replicates per condition were processed. The initial step involved collecting the samples and extracting RNA using the Roboklon RNA extraction kits, described in detail in Section 2.6. The quality of the extracted RNA was assessed using a Nanodrop spectrophotometer. Subsequent procedures were conducted at BGI Genomics, Budapest, Hungary. Libraries were prepared from total RNA using a stranded poly(A)-selected protocol: poly(A)^+^ RNA was enriched, fragmented, reverse-transcribed into cDNA, adapter-ligated, PCR-amplified, and quality-controlled. Libraries were sequenced on the DNBSEQ-G400 platform in paired-end mode with a read length of 150 bp (PE150), yielding approximately 50 million paired-end reads per sample. Most samples produced more than 50 million reads per end, with a small number yielding approximately 45–47 million reads per end. RNA-Seq reads were mapped to the human genome (GRCh38, v25) with STAR [30] (v-2.7.3a) using the following parameters:

--outSAMunmapped Within --outFilterType BySJout --outFilterMultimapNmax 20 --alignSJoverhangMin 8 --alignSJDBoverhangMin 1 --outFilterMismatchNmax 999 --outFilterMismatchNoverLmax 0.04 --alignIntronMin 20 --alignIntronMax 1000000 --alignMatesGapMax 1000000

Mapping efficiency was high, with most samples showing more than 90% reads mapped to GRCh38 (typically around 95%). Reads were assigned to genes using FeatureCounts [31] (v-2.0.6) with the following parameters: -T 2 -t exon -g gene_id -s 2 -p (the -p flag denotes paired-end reads. -s 2 denotes a reverse-stranded library). For the differential expression analyses we used DESeq2 [32] (version 1.38.0), following the canonical workflow of Love, Huber, and Anders (2014) with median-of-ratios size-factor normalization, the Wald test, and Benjamini–Hochberg correction for multiple testing. Genes with fewer than 5 counts in at least 3 samples were removed from the analysis prior to differential testing. The design formula was ~condition, where condition is a single factor whose levels encode the {cell line, temperature, treatment, time-point} combination of each sample (e.g., SW620_42_AMRF_0, U138_42_WB_RT_24). Each cell line was analysed in a separate DESeq2 dataset, and pairwise contrasts were extracted via the results() function (e.g., results(dds, contrast = c("condition","SW620_42_AMRF_0","SW620_42_WB_0"))).

No batch term was included in the DESeq2 design formula and no explicit batch correction was applied. Sequencing was performed at BGI Genomics (Budapest, Hungary). Sample preparation was distributed over the experimental window rather than a single date, and no formal correction for sample-preparation date or other potential batch sources was applied beyond DESeq2’s median-of-ratios size-factor normalization. Differentially expressed genes were defined as $|\log_{2}\mathrm{FC}|>1$ and Benjamini–Hochberg adjusted p<0.05.

Gene-set enrichment analysis was performed with the R/tmod package v-0.50.13 [33] using MSigDB-derived gene-set collections (Gene Ontology Biological Processes, KEGG pathways, and Reactome pathways). The reference gene universe was the set of all genes retained after the DESeq2 count-filter (∼24,000 genes per cell-line dataset). Module-level significance thresholds were AUC >0.7 and Benjamini–Hochberg FDR <0.05. The gene-level overlays used to color up- and down-regulated fractions in the tmod panel plots were drawn at $|\log_{2}\mathrm{FC}|>1$ and adjusted *p*-value <0.05.

### 2.8. Statistical Analysis

**Figure 1:**
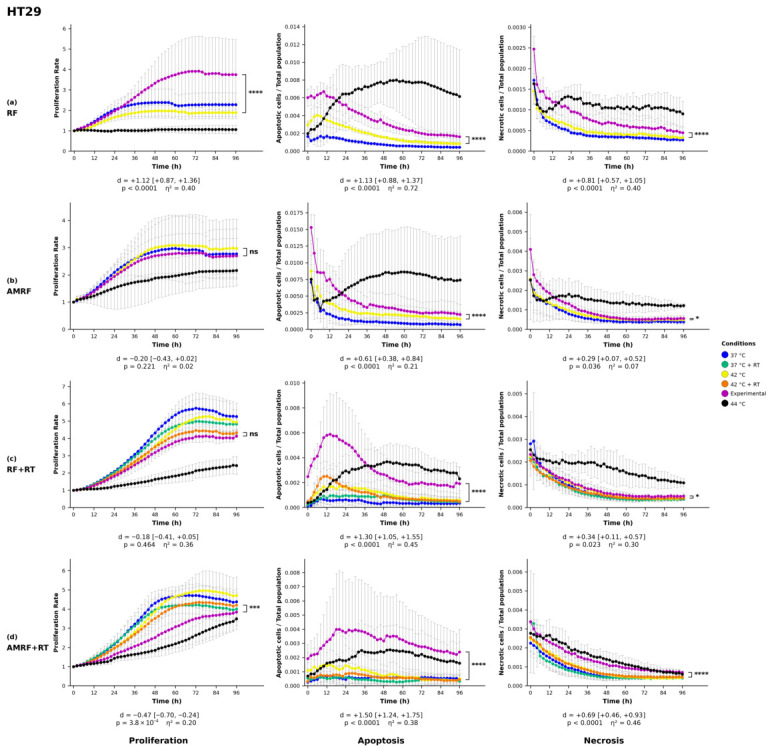
Phenotypic profiling of HT29 colorectal cancer cells following RF-based and control treatments. Real-time monitoring of proliferation, apoptosis, and necrosis was conducted over a 96 h period using live-cell imaging in response to (**a**) radiofrequency (RF), (**b**) amplitude-modulated RF (AMRF), (**c**) RF combined with radiotherapy (RF+RT), and (**d**) AMRF combined with radiotherapy (AMRF+RT). Each experimental condition was compared exclusively to its corresponding water bath (WB) control at 42 °C. Plots display proliferation (left), apoptosis (Annexin V, middle), and necrosis (YOYO-3, right) for each treatment (rows). Data are expressed as mean ± standard deviation (SD) from n=3 independent biological replicates per condition, each analysed in technical triplicate (3×3 design). Error bars are rendered as T-capped bars. The y-axes show normalized confluency (proliferation, fold-change relative to t=0), Annexin V^+^ object count per well (apoptosis), and YOYO-3^+^ object count per well (necrosis). The x-axis is time post-exposure (h). Statistical analysis was performed using two-way analysis of variance (ANOVA) with condition and time as factors, followed by Šídák’s multiple-comparisons post hoc test applied to the pre-planned family of experimental vs. each control contrasts. Only the experimental vs. matched-42 °C (±RT)-WB contrast is displayed. Confluency values were normalized to the t=0 reading per well. Asterisks indicate statistical significance relative to the matched 42 °C (±RT) WB control: * p<0.05, *** p<0.001, **** p<0.0001, ns = not significant. Below each panel, a two-line strip reports the Hedges-corrected Cohen’s *d* with 95% confidence interval (upper line) and the Šídák-adjusted *p*-value with partial $\eta^{2}$ for the condition main effect (lower line). The y-axis lower bound is fixed at zero, since the reported quantities cannot be negative. SD whiskers extending below this bound are clipped at the axis.

**Figure 2:**
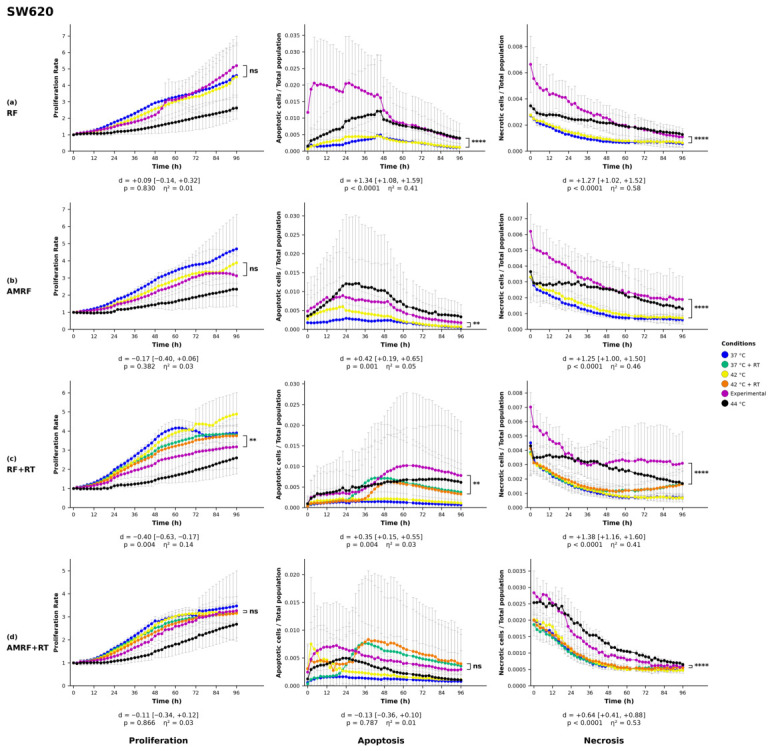
Phenotypic profiling of SW620 colorectal cancer cells following RF-based and control treatments. Real-time monitoring of proliferation, apoptosis, and necrosis was conducted over a 96 h period using live-cell imaging in response to (**a**) radiofrequency (RF), (**b**) amplitude-modulated RF (AMRF), (**c**) RF combined with radiotherapy (RF+RT), and (**d**) AMRF combined with radiotherapy (AMRF+RT). Each experimental condition was compared exclusively to its corresponding water bath (WB) control at 42 °C. For non-irradiated groups, comparisons were made to 42 °C WB alone. For irradiated groups, comparisons were made to 42 °C + RT WB. Plots display proliferation (left), apoptosis (Annexin V, middle), and necrosis (YOYO-3, right) for each treatment (rows). Data are expressed as mean ± standard deviation (SD) from n=3 independent biological replicates per condition, each analyzed in technical triplicate (3×3 design). Error bars are rendered as T-capped bars. The y-axes show normalized confluency (proliferation, fold-change relative to t=0), Annexin V^+^ object count per well (apoptosis), and YOYO-3^+^ object count per well (necrosis). The x-axis is time post-exposure (h). Statistical analysis was performed using two-way analysis of variance (ANOVA) with condition and time as factors, followed by Šídák’s multiple-comparisons post hoc test applied to the pre-planned family of experimental vs. each control contrasts. Only the experimental vs. matched-42 °C (±RT)-WB contrast is displayed. Confluency values were normalized to the t=0 reading per well. Asterisks indicate statistical significance relative to the matched 42 °C (±RT) WB control: ** p<0.01, **** p<0.0001, ns = not significant. Below each panel, a two-line strip reports the Hedges-corrected Cohen’s *d* with 95% confidence interval (upper line) and the Šídák-adjusted *p*-value with partial $\eta^{2}$ for the condition main effect (lower line). The y-axis lower bound is fixed at zero, since the reported quantities cannot be negative. SD whiskers extending below this bound are clipped at the axis.

**Figure 3:**
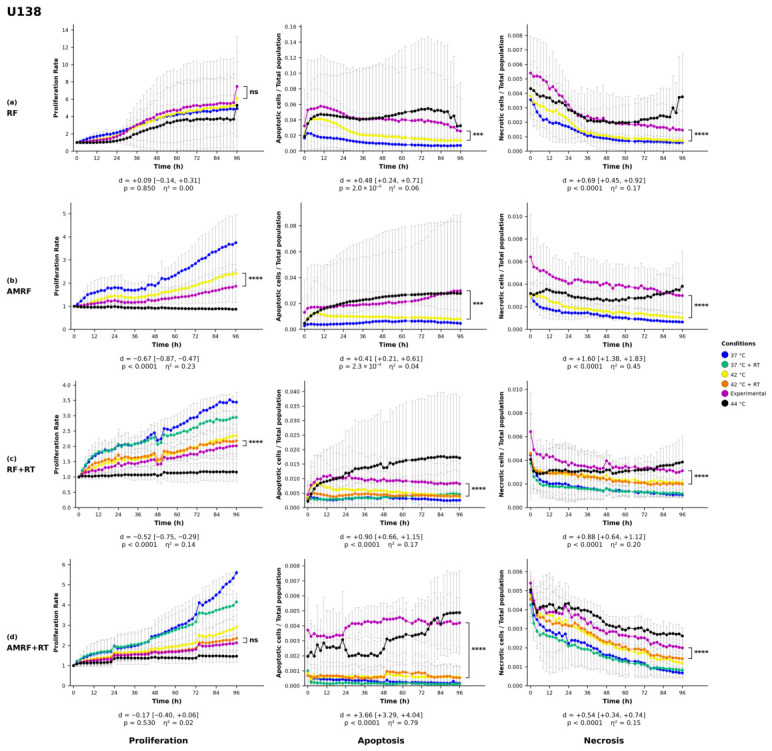
Phenotypic responses of U138 glioblastoma cells to RF-based and control treatments. Real-time monitoring of proliferation, apoptosis, and necrosis was conducted over a 96 h period using live-cell imaging in response to (**a**) radiofrequency (RF), (**b**) amplitude-modulated RF (AMRF), (**c**) RF combined with radiotherapy (RF+RT), and (**d**) AMRF combined with radiotherapy (AMRF+RT). Each treatment condition was compared exclusively to its corresponding 42 °C water bath (WB) control. For non-irradiated treatments, comparisons were made against 42 °C WB, while irradiated conditions were compared against the 42 °C + RT WB group. Plots display proliferation (left), apoptosis (Annexin V, middle), and necrosis (YOYO-3, right) for each treatment (rows). Data are expressed as mean ± standard deviation (SD) from n=3 independent biological replicates per condition, each analyzed in technical triplicate (3×3 design). Error bars are rendered as T-capped bars. The y-axes show normalized confluency (proliferation, fold-change relative to t=0), Annexin V^+^ object count per well (apoptosis), and YOYO-3^+^ object count per well (necrosis). The x-axis is time post-exposure (h). Statistical analysis was performed using two-way analysis of variance (ANOVA) with condition and time as factors, followed by Šídák’s multiple-comparisons post hoc test applied to the pre-planned family of experimental vs. each control contrasts. Only the experimental vs. matched-42 °C (±RT)-WB contrast is displayed. Confluency values were normalized to the t=0 reading per well. Asterisks indicate significant differences relative to the corresponding 42 °C or 42 °C + RT control: *** p<0.001, **** p<0.0001, ns = not significant. Below each panel, a two-line strip reports the Hedges-corrected Cohen’s *d* with 95% confidence interval (upper line) and the Šídák-adjusted *p*-value with partial $\eta^{2}$ for the condition main effect (lower line). The y-axis lower bound is fixed at zero, since the reported quantities cannot be negative. SD whiskers extending below this bound are clipped at the axis.

**Figure 4:**
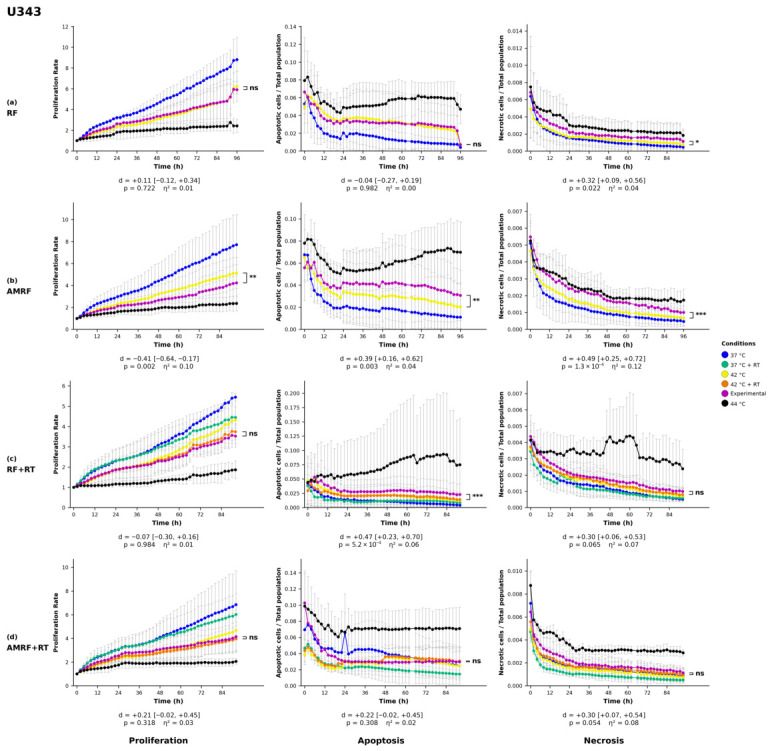
Phenotypic responses of U343 glioblastoma cells to RF-based and control treatments. Real-time monitoring of proliferation, apoptosis, and necrosis was conducted over a 96 h period using live-cell imaging in response to (**a**) radiofrequency (RF), (**b**) amplitude-modulated RF (AMRF), (**c**) RF combined with radiotherapy (RF+RT), and (**d**) AMRF combined with radiotherapy (AMRF+RT). Each treatment condition was compared exclusively to its corresponding 42 °C water bath (WB) control. For non-irradiated treatments, comparisons were made against 42 °C WB, while irradiated conditions were compared against the 42 °C + RT WB group. Plots display proliferation (left), apoptosis (Annexin V, middle), and necrosis (YOYO-3, right) for each treatment (rows). Data are expressed as mean ± standard deviation (SD) from n=3 independent biological replicates per condition, each analysed in technical triplicate (3×3 design). Error bars are rendered as T-capped bars. The y-axes show normalized confluency (proliferation, fold-change relative to t=0), Annexin V^+^ object count per well (apoptosis), and YOYO-3^+^ object count per well (necrosis). The x-axis is time post-exposure (h). Statistical analysis was performed using two-way analysis of variance (ANOVA) with condition and time as factors, followed by Šídák’s multiple-comparisons post hoc test applied to the pre-planned family of experimental vs. each control contrasts. Only the experimental vs. matched-42 °C (±RT)-WB contrast is displayed. Confluency values were normalized to the t=0 reading per well. Asterisks indicate significant differences relative to the corresponding 42 °C or 42 °C + RT control: * p<0.05, ** p<0.01, *** p<0.001, ns = not significant. Below each panel, a two-line strip reports the Hedges-corrected Cohen’s *d* with 95% confidence interval (upper line) and the Šídák-adjusted *p*-value with partial $\eta^{2}$ for the condition main effect (lower line). The y-axis lower bound is fixed at zero, since the reported quantities cannot be negative. SD whiskers extending below this bound are clipped at the axis.

All statistics on proliferation, apoptosis, and necrosis were performed by two-way analysis of variance (ANOVA) with treatment condition and imaging time point as factors, followed by Šídák’s multiple-comparisons post hoc test applied to the pre-planned family of experimental vs. each control pairwise contrasts (three comparisons for non-irradiated treatments, five for irradiated treatments). Each experimental condition (RF, AMRF, RF+RT, AMRF+RT) was compared against its matched water-bath (WB) control—42 °C for non-irradiated treatments and 42 °C + RT for irradiated treatments—and only this pairwise contrast is displayed in Figure 1, Figure 2, Figure 3 and Figure 4. Šídák’s correction was chosen over Tukey’s HSD because our pre-planned comparison family comprises only experimental vs. each control contrasts and does not include uninformative controls vs. controls pairings, for which Tukey would impose unnecessary correction. Confluency values were first normalized to each well’s value at t=0 to correct for inter-well seeding-density variation. Statistical analyses were performed in Python 3.14 (scipy 1.17.1, statsmodels 0.14.6). Two-tailed *p*-values below 0.05 were defined as statistically significant (* = p<0.05, ** = p<0.01, *** = p<0.001, **** = p<0.0001).

## 3. Results

### 3.1. Proliferation, Apoptosis, and Necrosis Rates

Two colorectal cancer (HT29, SW620) and two glioblastoma (U138, U343) cell lines were exposed to RF, AMRF, and their combinations with RT. Proliferation, apoptosis, and necrosis profiles are reported below. Transcriptomic profiling in SW620 and U138 follows in Section 3.2.

#### 3.1.1. Colorectal Cancer Cell Lines

In the HT29 cell line (Figure 1), RF alone led to a statistically significant increase in proliferation compared to the controls (p<0.0001), while AMRF+RT significantly reduced proliferation (p<0.001). AMRF and RF+RT did not induce statistically significant changes in proliferation relative to their matched thermal controls. Apoptosis was markedly elevated across all treatment conditions (p<0.0001), with the combinational therapies RF+RT and AMRF+RT inducing even higher apoptotic rates, not only in comparison to the 42 °C WB control but also surpassing those of the 44 °C WB control, suggesting an enhanced apoptotic effect. Necrosis was significantly increased in all treatment groups, with RF and AMRF+RT demonstrating p<0.0001, while AMRF and RF+RT reached p<0.05, indicating a consistent necrosis response across conditions.

In the SW620 cell line (Figure 2), proliferation remained largely unaffected, with the exception of the RF+RT condition, which led to a statistically significant reduction (p<0.01). Apoptosis was markedly elevated following RF treatment (p<0.0001), while AMRF and RF+RT induced a statistically significant increase (p<0.01). Conversely, the AMRF+RT condition did not yield a statistically significant apoptotic response. Necrosis was markedly induced across all treatment conditions (p<0.0001), with necrosis rates in all experimental groups consistently exceeding those observed in all control groups, including the 44 °C WB control, indicating a consistent necrosis response across RF- and AMRF-based treatments.

#### 3.1.2. Glioblastoma Cell Lines

In the U138 glioblastoma cell line (Figure 3), proliferation was significantly reduced in response to AMRF and RF+RT (p<0.0001), whereas no statistically significant effects were observed for RF or AMRF+RT. Apoptosis was markedly increased following RF and AMRF (p<0.001) and further elevated in the combinational therapies RF+RT and AMRF+RT (p<0.0001), suggesting enhanced apoptotic activity when RF-based therapies were combined with radiation. Necrosis was markedly induced across all treatment conditions (p<0.0001), indicating a consistent increase in necrosis-mediated cell death irrespective of the applied RF-based therapy.

In the U343 glioblastoma cell line (Figure 4), proliferation was significantly reduced only by AMRF (p<0.01), while the remaining treatment conditions (RF, RF+RT, and AMRF+RT) did not lead to statistically significant changes. Apoptosis was moderately elevated in response to AMRF (p<0.01) and markedly increased following RF+RT (p<0.001), whereas the RF and AMRF+RT conditions did not elicit a statistically significant apoptotic response. Necrosis was significantly increased following RF treatment (p<0.05), with a more pronounced effect observed for AMRF (p<0.001). The RF+RT and AMRF+RT conditions did not induce a significant necrosis response, indicating cell-line-specific necrosis patterns in U343 compared to U138 cells.

### 3.2. RNA-Seq Analysis in Treated Cells

Based on the initial phenotypic assessments (apoptosis, necrosis, and proliferation), RNA sequencing was selectively conducted on the SW620 colorectal and U138 glioblastoma cell lines, which demonstrated the most pronounced and differential responses to treatment. These two models were thus chosen for deeper transcriptomic investigation. For each condition, samples were collected at two distinct time points to capture both immediate and delayed transcriptional responses: the “0 h” samples refer to RNA extracted directly after treatment, while the “24 h” samples represent cells that were re-seeded in petri dishes and incubated under standard conditions for 24 h before RNA extraction. This dual time-point approach enabled the investigation of both early cellular signaling and sustained gene expression dynamics following RF- and AMRF-based therapies.

#### 3.2.1. Impact of Reference Therapies: RT and HT

**Figure 5:**
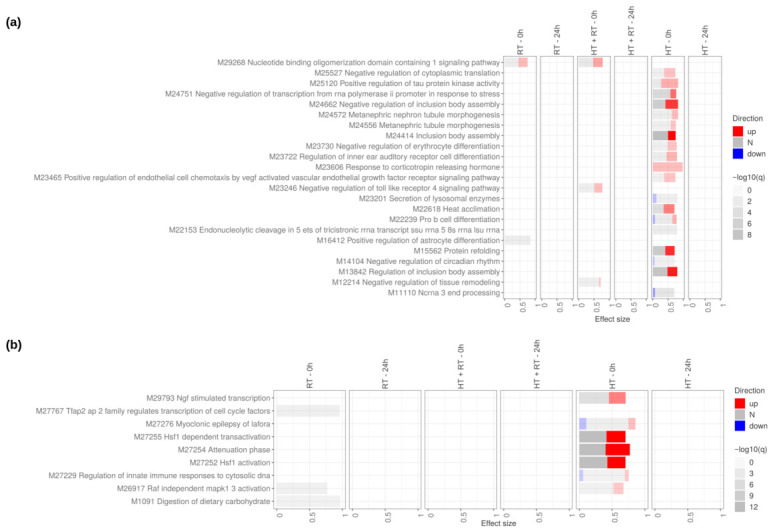
Impact of reference therapies on SW620 cells at 0 h and 24 h. Tmod [33] panel plots showing gene-set enrichment analysis using Molecular Signatures Database (MSigDB) gene sets for (**a**) Gene Ontology Biological Processes (GO:BP) and (**b**) Reactome pathways. The analyses compare the effects of reference therapies (HT, RT, and HT+RT) to untreated controls at 0 h and 24 h. The size of each rectangle is the effect size (AUC). Red and blue denote the fraction of genes significantly up- or down-regulated by more than two-fold, and grey (N) marks gene sets that are significantly enriched but without a predictable direction of change. Cutoffs were gene $|\log_{2}\mathrm{FC}|>1$, gene adjusted p<0.05, gene-set FDR <0.05, and AUC >0.7. Ontologies with no significant gene set are not shown.

For SW620, transcriptome-wide differential expression analysis (Figure 5, Supplementary Table S1, Supplementary Figure S1) showed that HT caused the earliest and most extensive transcriptional response, whereas RT alone had relatively minor effects, and HT+RT produced an intermediate phenotype. While RT exposure led to only small transcriptional shifts with relatively few genes reaching high statistical significance, HT triggered a broad transcriptional reprogramming across multiple functional modules, including stress adaptation, vascular remodeling, immune modulation, cell cycle activation, and extracellular matrix remodeling.

At 0 h post-HT, stress-response pathways such as HSF1 activation, HSF1-dependent transactivation (Figure 5b), and protein refolding (Figure 5a) were significantly up-regulated, indicating activation of the canonical heat-shock response and protein homeostasis mechanisms. Vascular remodeling pathways, including VEGF-mediated endothelial chemotaxis, were also significantly modulated.

Interestingly, immune and inflammatory signaling showed a dual pattern, with suppression of acute pro-inflammatory cascades such as Toll-like receptor signaling and transient activation of immune differentiation pathways, including Pro-B cell differentiation. A shift toward immune modulation rather than broad activation is suggested. By 24 h post-treatment, transcriptional changes in all modules largely subsided, showing that HT-induced cellular reprogramming is immediate but reversible, with most signaling events peaking instantly after exposure and returning toward baseline within one day.

**Figure 6:**
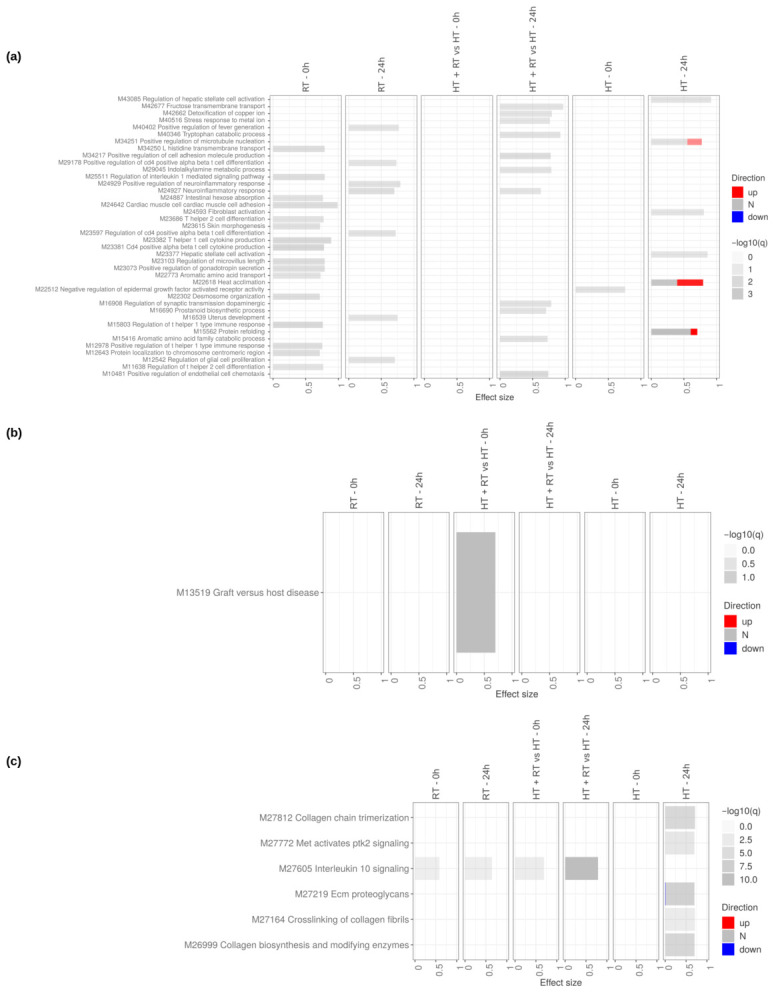
Impact of reference therapies on U138 cells at 0 h and 24 h. Tmod [33] panel plots showing gene set enrichment analysis using MSigDB gene sets for (**a**) Biological Processes and (**b**) KEGG and (**c**) Reactome pathways. The analyses compare the effects of reference therapies (HT, RT, and HT+RT) to untreated controls at 0 h and 24 h. The size of each rectangle is the effect size (AUC). Red and blue denote the fraction of genes significantly up- or down-regulated by more than two-fold, and grey (N) marks gene sets that are significantly enriched but without a predictable direction of change. Cutoffs were gene $|\log_{2}\mathrm{FC}|>1$, gene adjusted p<0.05, gene-set FDR <0.05, and AUC >0.7. Ontologies with no significant gene set are not shown.

In contrast to SW620 colorectal cancer cells, which displayed broad and multi-layered transcriptional reprogramming across all treatments, U138 glioblastoma cells exhibited a far more limited and selective response (Figure 6, Supplementary Table S2, Supplementary Figure S4). Transcriptomic profiling revealed that HT induced a broader and temporally more dynamic transcriptional response than RT, with changes primarily emerging at the 24 h timepoint. RT exposure led to only minimal transcriptional shifts at both 0 h and 24 h, with very few pathways showing significant modulation, indicating a restricted and low-amplitude cellular response. In contrast, HT triggered a wider spectrum of transcriptional alterations, particularly at 24 h, suggesting that thermal stress engages additional cellular programs over time, even though these changes remain limited compared to those in SW620 cells.

Functional enrichment analysis across GO Biological Processes (Figure 6a), KEGG (Figure 6b), and Reactome (Figure 6c) showed that RT caused insignificant effects on most pathways. By contrast, HT at 24 h led to important modulation of pathways related to extracellular matrix (ECM) organization, vascular remodeling, and immune regulation, including collagen biosynthesis and modifying enzymes, crosslinking of collagen fibrils, ECM proteoglycans, fibroblast activation, endothelial cell chemotaxis, and Interleukin-10 signaling. However, the orientation of these changes was mixed, with some pathways showing evidence of suppression rather than activation, indicating that HT primarily alters rather than uniformly induces these processes. HT+RT did not amplify these effects, suggesting that in U138 cells, HT alone, rather than its combination with RT, drives the limited transcriptional reprogramming observed at 24 h, while RT itself remains largely transcriptionally inactive.

#### 3.2.2. Impact of RF and AMRF and Their Combination with RT

Transcriptomic profiling in SW620 cells revealed that AMRF elicited the most extensive and dynamic transcriptional reprogramming among all treatments, with changes peaking immediately after exposure (0 h) and diminishing by 24 h, whereas RF and RF+RT showed only modest effects (Supplementary Table S3, Supplementary Figure S2). AMRF+RT displayed an intermediate phenotype, showing broader regulatory activity than RF+RT but less than AMRF alone, as quantified by direct treatment vs. treatment contrasts (Supplementary Table S4, Supplementary Figure S3).

**Figure 7:**
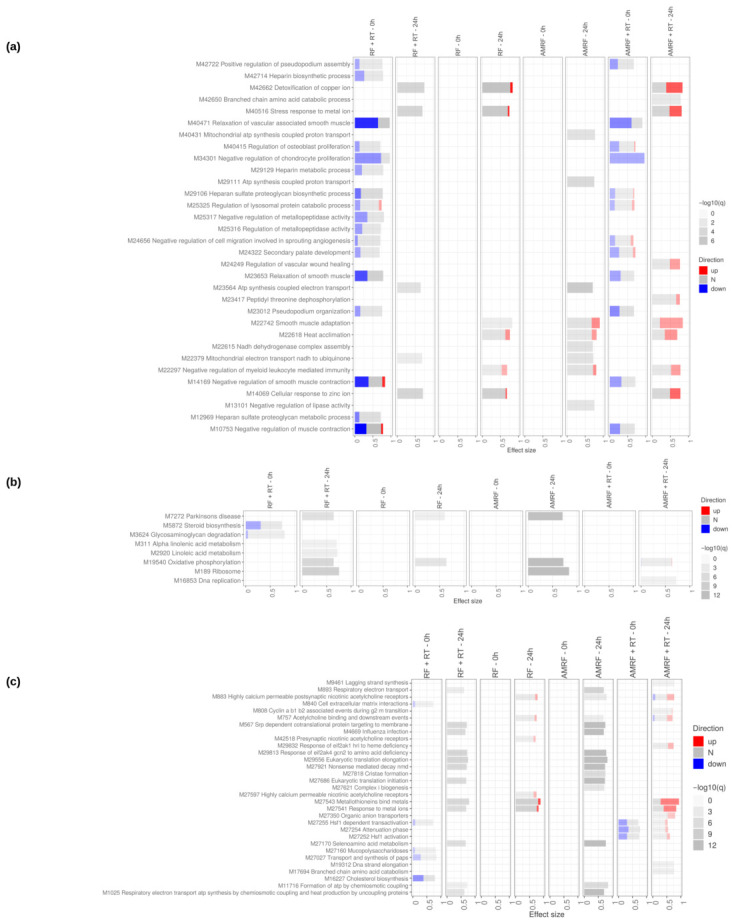
Impact of RF, AMRF and their combination with RT on SW620 cells at 0 h and 24 h. Tmod [33] panel plots showing gene set enrichment analysis using MSigDB gene sets for (**a**) Biological Processes and (**b**) KEGG and (**c**) Reactome pathways. The analyses compare the effects of RF and AMRF therapies as well as their combination with RT to controls only treated with HT and HT+RT respectively, at 0 h and 24 h. The size of each rectangle is the effect size (AUC). Red and blue denote the fraction of genes significantly up- or down-regulated by more than two-fold, and grey (N) marks gene sets that are significantly enriched but without a predictable direction of change. Cutoffs were gene $|\log_{2}\mathrm{FC}|>1$, gene adjusted p<0.05, gene-set FDR <0.05, and AUC >0.7. Ontologies with no significant gene set are not shown. For the Biological Processes panel (**a**), a stricter gene-set FDR <0.01 was used to limit the number of displayed pathways.

When RF- and AMRF-based regimens were directly compared against heat controls (HT or HT+RT) (Figure 7), the data revealed that both RF and AMRF triggered additional transcriptional paths apart from those induced by HT itself. AMRF-containing regimens, particularly AMRF+RT at 24 h, exhibited the strongest additive effects, where processes related to metal detoxification, mitochondrial respiration, and oxidative phosphorylation were consistently enriched. These findings indicate that AMRF, especially in combination with RT, amplifies the canonical heat-shock response with metabolic and redox adaptation programs not observed under heat alone.

**Figure 8:**
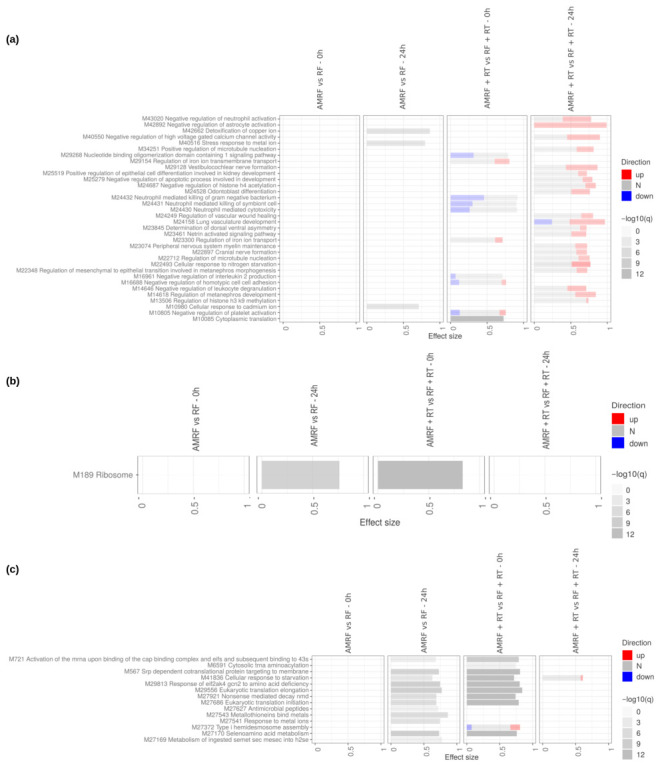
Direct comparison of the impact of AMRF and AMRF+RT to RF and RF+RT respectively, on SW620 cells at 0 h and 24 h. Tmod [33] panel plots showing gene set enrichment analysis using MSigDB gene sets for (**a**) Biological Processes and (**b**) KEGG and (**c**) Reactome pathways. The size of each rectangle is the effect size (AUC). Red and blue denote the fraction of genes significantly up- or down-regulated by more than two-fold, and grey (N) marks gene sets that are significantly enriched but without a predictable direction of change. Cutoffs were gene $|\log_{2}\mathrm{FC}|>1$, gene adjusted p<0.05, gene-set FDR <0.05, and AUC >0.7. Ontologies with no significant gene set are not shown.

The evidence (Figure 7 and Figure 8) further highlighted that AMRF-containing regimens, but not RF alone, engaged amino acid starvation signaling and eIF2-mediated stress responses, suggesting an adaptive mechanism observed specifically in AMRF arms in SW620 cells.

**Figure 9:**
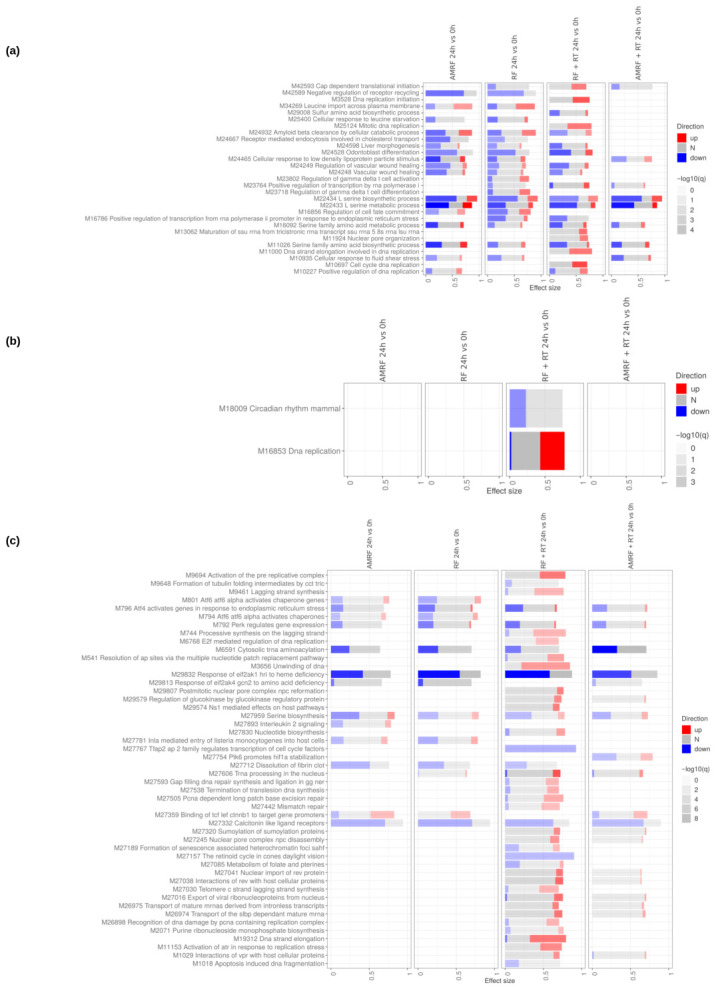
Direct comparison of the RF and AMRF-containing regimens’ timepoints in SW620 cells. Tmod [33] panel plots showing gene set enrichment analysis using MSigDB gene sets for (**a**) Biological Processes and (**b**) KEGG and (**c**) Reactome pathways. The size of each rectangle is the effect size (AUC). Red and blue denote the fraction of genes significantly up- or down-regulated by more than two-fold, and grey (N) marks gene sets that are significantly enriched but without a predictable direction of change. Cutoffs were gene $|\log_{2}\mathrm{FC}|>1$, gene adjusted p<0.05, gene-set FDR <0.05, and AUC >0.7. Ontologies with no significant gene set are not shown. For the Biological Processes panel (**a**), a stricter gene-set FDR <0.01 was used to limit the number of displayed pathways.

Direct timepoint comparisons (24 h vs. 0 h) (Figure 9) showed that cell cycle re-entry, DNA replication, and DNA repair pathways became enriched in RT-containing arms (RF+RT and AMRF+RT), consistent with the engagement of DNA-damage-response programs at 24 h post-irradiation. The non-irradiated AMRF and RF arms showed instead a coherent down-regulation of ER-stress-response pathways (ATF4, ATF6, PERK signaling) at 24 h, indicating a return toward homeostasis after the acute response.

Across all modules, AMRF induced the broadest and most coherent transcriptional reprogramming relative to heat alone, integrating stress adaptation, mitochondrial activation, vascular remodeling, ECM reorganization, cell cycle progression, and protein synthesis. AMRF+RT amplified selected stress and metabolic programs but remained less extensive than AMRF alone, while RF- and RF+RT-specific effects were weaker, delayed, and mainly inflammation-centered.

Overall, these findings show that the additional effects of RF- and AMRF-based treatments beyond HT alone are dominated by AMRF, which uniquely combines fast stress adaptation with metabolic and vascular remodeling signatures, whereas RF boosts transient immune and inflammatory pathways without broader cellular reprogramming.

**Figure 10:**
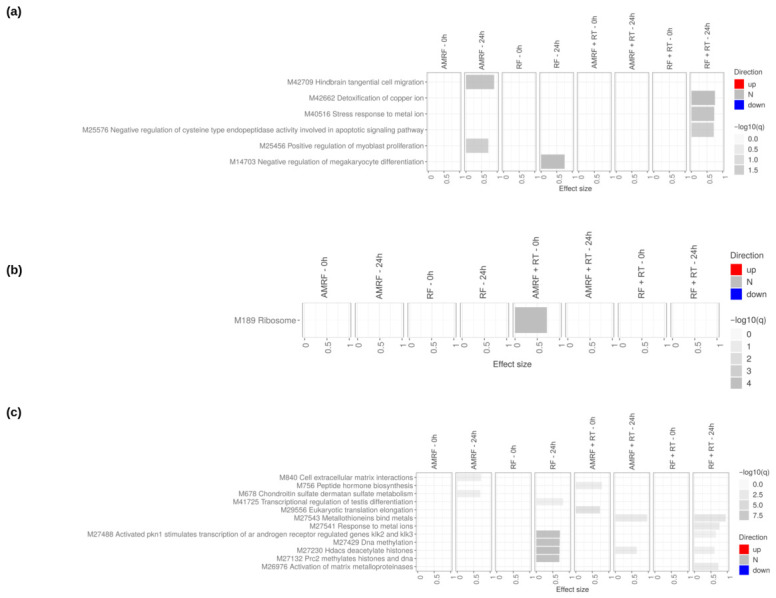
Impact of RF, AMRF, and their combination with RT on U138 cells at 0 h and 24 h. Tmod [33] panel plots showing gene set enrichment analysis using MSigDB gene sets for (**a**) Biological Processes and (**b**) KEGG and (**c**) Reactome pathways. The analyses compare the effects of RF and AMRF therapies as well as their combination with RT to controls only treated with HT and HT+RT respectively, at 0 h and 24 h. The size of each rectangle is the effect size (AUC). Red and blue denote the fraction of genes significantly up- or down-regulated by more than two-fold, and grey (N) marks gene sets that are significantly enriched but without a predictable direction of change. Cutoffs were gene $|\log_{2}\mathrm{FC}|>1$, gene adjusted p<0.05, gene-set FDR <0.05, and AUC >0.7. Ontologies with no significant gene set are not shown.

Transcriptomic profiling in U138 glioblastoma cells revealed a markedly attenuated response to all interventions compared to SW620 cells, with both the RF/AMRF vs. HT reference contrasts (Supplementary Table S5, Supplementary Figure S5) and the direct AMRF vs. RF and AMRF+RT vs. RF+RT comparisons (Supplementary Table S6, Supplementary Figure S6) showing a cell-type-specific difference in transcriptional sensitivity. Using HT as the reference, both early (0 h) and late (24 h) comparisons (Figure 10) showed that RF and AMRF added little beyond the transcriptional changes already induced by heat alone. Modest and inconsistent enrichment was detectable across the four arms, with chromatin-modification pathways (DNA methylation, histone deacetylation, PRC2-mediated methylation) appearing in RF at 24 h and metal-ion detoxification appearing in RF+RT at 24 h, but these changes remained limited in amplitude and did not present consistent patterns of activation or suppression, indicating only limited engagement of adaptive responses.

**Figure 11:**
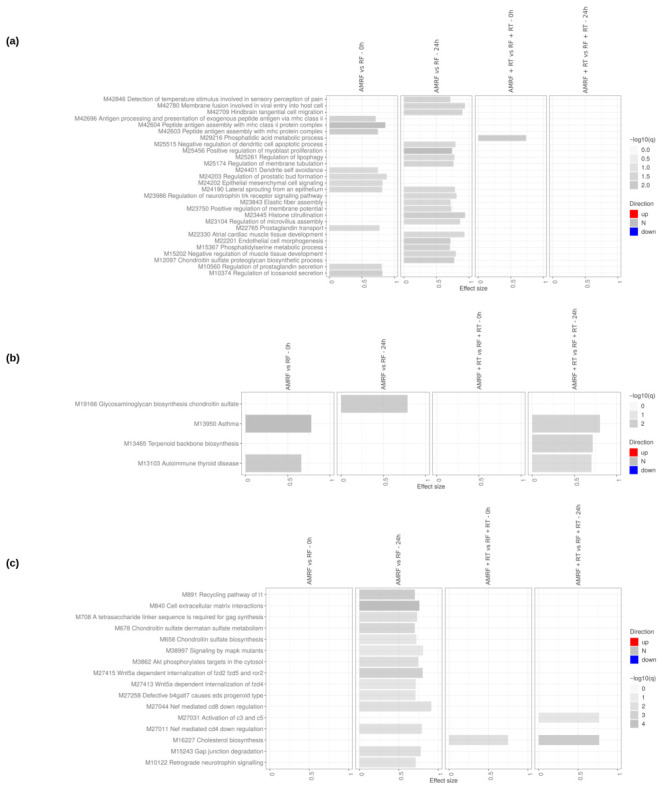
Direct comparison of the impact of AMRF and AMRF+RT to RF and RF+RT respectively, on U138 cells at 0 h and 24 h. Tmod [33] panel plots showing gene set enrichment analysis using MSigDB gene sets for (**a**) Biological Processes and (**b**) KEGG and (**c**) Reactome pathways. The size of each rectangle is the effect size (AUC). Red and blue denote the fraction of genes significantly up- or down-regulated by more than two-fold, and grey (N) marks gene sets that are significantly enriched but without a predictable direction of change. Cutoffs were gene $|\log_{2}\mathrm{FC}|>1$, gene adjusted p<0.05, gene-set FDR <0.05, and AUC >0.7. Ontologies with no significant gene set are not shown.

Direct comparisons (Figure 11) between AMRF and RF, as well as AMRF+RT and RF+RT, confirmed this overall picture. AMRF at 24 h vs. RF showed broader low-intensity enrichment across extracellular matrix interactions, chondroitin sulfate biosynthesis and metabolism, glycosaminoglycan biosynthesis, antigen presentation, MAPK signaling, and Wnt5a-dependent receptor internalization. Combination treatments at 24 h (AMRF+RT vs. RF+RT) instead showed a narrower additional signature dominated by cholesterol biosynthesis, terpenoid backbone biosynthesis, and complement (C3/C5) activation. Across all comparisons, signals remained weak and lacked the coordinated up- or down-regulation observed in SW620 cells.

**Figure 12:**
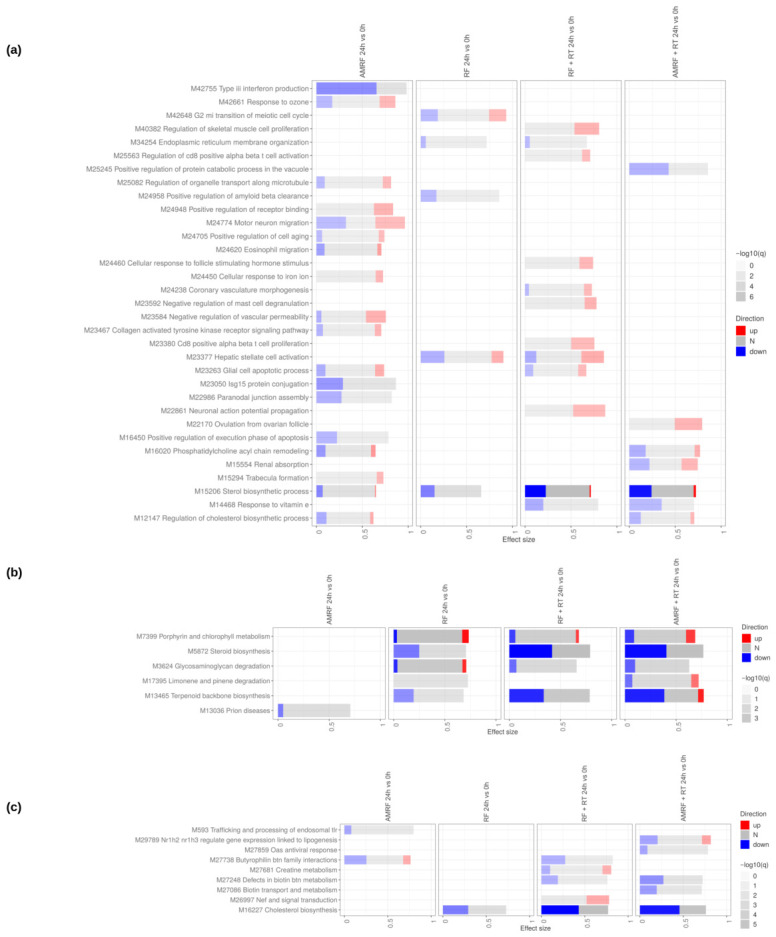
Direct comparison of the RF and AMRF-containing regimens’ timepoints in U138 cells. Tmod [33] panel plots showing gene set enrichment analysis using MSigDB gene sets for (**a**) Biological Processes and (**b**) KEGG and (**c**) Reactome pathways. The size of each rectangle is the effect size (AUC). Red and blue denote the fraction of genes significantly up- or down-regulated by more than two-fold, and grey (N) marks gene sets that are significantly enriched but without a predictable direction of change. Cutoffs were gene $|\log_{2}\mathrm{FC}|>1$, gene adjusted p<0.05, gene-set FDR <0.05, and AUC >0.7. Ontologies with no significant gene set are not shown.

Time-resolved analyses (24 h vs. 0 h) (Figure 12) further demonstrated that transcriptional changes in U138 cells were generally limited in magnitude. Scattered modulation of cytokine signaling and metabolic processes was detectable, with a consistent down-regulation of sterol and cholesterol biosynthesis at 24 h across RT-containing arms.

Overall, this integrated analysis shows that when assessed relative to heat controls, RF- and AMRF-based treatments elicit only weak and short-lived transcriptional effects in U138 cells, even under direct method comparisons and temporal profiling. This comes in contrast with SW620 cells, where AMRF triggered broad, multi-layered, and time-coordinated transcriptional reprogramming, highlighting a pronounced cell-type-specific divergence in treatment sensitivity.

## 4. Discussion

To date, the non-temperature-induced effects of AMRF have been underexplored. Earlier studies largely emphasized on theoretical models such as ion channel-mediated membrane effects [9], tumor-specific modulation frequencies [12], AMRF-induced tumor damage mechanisms [34], or clinical survival benefits reported for regional HT and AMRF [10,13,35], but lacked systematic integration of phenotypic and transcriptomic evidence.

A central interpretive question for any study of this kind is whether the observed RF- and AMRF-specific effects could be explained by hidden thermal gradients rather than by genuinely non-temperature-induced energy-deposition mechanisms. Three observations argue against a hidden-thermal explanation. First, all RF and AMRF arms were compared against matched-temperature water-bath (WB) controls at 37 °C and 42 °C (±RT), in which the cell suspension experiences the same bulk temperature without any RF field. Treatment-specific effects observed relative to these matched-temperature WB controls cannot, by construction, have a purely thermal origin. Second, in-suspension temperature was monitored throughout each exposure with the same fluoroptic sensor in both the LabEHY-200 chamber and the water-bath control tubes (Section 2.1 and Section 2.4). Third, as quantified in Section 2.1, a local SAR of 10,000 W/kg focused into a 1 mm radius sphere would produce a maximum temperature excursion of only 2 °C (refs. [3,9]). Most directly, AMRF+RT produced necrotic responses exceeding those of the 44 °C WB positive-control condition in HT29 (Figure 1) and SW620 (Figure 2): the biological effect is greater than that of an unequivocally supra-thermal control, which is incompatible with a hidden-thermal-gradient explanation.

Our findings are consistent with the ion channel-mediated membrane model previously proposed by our group [9]: therapeutic-level AMRF exhibits transcriptomic signatures that are the expected downstream consequences of non-temperature-induced ionic perturbations (e.g., Ca^2+^ influx/K^+^ efflux), with a 24 h up-regulation of mitochondrial/oxidative phosphorylation pathways, redox-adaptation pathways, and metal-handling/detoxification modules (Cu/Zn, metallothioneins). This is a broadened stress response that surpasses the expected heat-shock response, and is consistent with a coherent two-step research arc on AMRF and the heat-shock axis: Danics et al. 2020 [36] showed that AMRF exhausts the protective heat-shock response in triple-negative breast cancer (TNBC) isografts using the same LabEHY-200 device employed in the present study, and Viana et al. 2024 [37] demonstrated that pharmacological HSF1 inhibition further potentiates AMRF efficacy in the same TNBC model. The SW620 transcriptomic activation we observe is consistent with the up-stream HSF1-axis engagement step of that arc, suggesting that HSF1 inhibition represents a candidate sensitiser that could be combined with AMRF in future preclinical work. Concomitant necrotic phenotypes and immune/ECM signaling fit a scenario in which ionic imbalance and oxidative burden exceed cellular buffering capacity [38].

AMRF, alone and in combination with RT, was associated with broader and more sustained biological effects than RF, HT, or RT alone in CRC cells, with concurrent activation of multi-layered programs (immune modulation, ECM remodeling, proliferative signaling) alongside consistent necrosis. These effects were not uniformly recapitulated in the GBM lines, where AMRF+RT produced the strongest apoptotic response in U138 but neither RF+RT nor AMRF+RT produced a significant necrotic response in U343 (Figure 3 and Figure 4). The CRC findings align with clinical AMRF studies reporting improved disease control without additional toxicity [10,13,35,39,40,41] and extend earlier RF research [2,8,9,12] by revealing temporally stratified molecular responses associated with amplitude modulation.

In contrast, U138 GBM cells displayed strong apoptotic and necrotic responses phenotypically but only weak transcriptional alterations. This phenotype–transcriptome divergence is a factual observation that points to mechanisms operating downstream of, or in parallel with, transcription. The present data do not directly identify which mechanism is responsible for this divergence. We therefore propose the following candidates as explicit, falsifiable hypotheses for dedicated follow-up studies, prioritized on the basis of our group’s two prior physical-characterization frameworks [3,9], rather than as conclusions established by this study.

**Lead hypothesis—ion-channel rectification and ionic disequilibrium** [9]. At the field strengths characterized for the sandwich in vitro applicator used here (Section 2.1), the framework of Ref. [9] predicts that the RF electric field is rectified by membrane ion channels, producing a persistent net DC voltage across the membrane that, integrated over channel density and exposure time, can drive ion fluxes (particularly intracellular Ca^2+^ overload) sufficient to trigger apoptosis and necrosis without requiring transcriptional reprogramming. GBM cells are known to exhibit altered oncochannel expression, including Kir4.1, TRPV4, and KCa3.1 [42,43,44,45], which would further amplify this effect. This mechanism is a candidate for the U138 phenotype–transcriptome divergence because it acts post-transcriptionally and applies to both RF and AMRF arms. *Testable predictions:* (a) Pharmacological blockade of candidate oncochannels (BaCl_2_ for Kir, TRAM-34 for KCa3.1, HC-067047 for TRPV4) prior to AMRF should reduce U138 apoptosis/necrosis in a channel-specific manner. (b) Fura-2 ratiometric intracellular Ca^2+^ imaging immediately following AMRF exposure (the closed sandwich applicator precludes real-time imaging during exposure) should reveal residual elevation of cytosolic Ca^2+^ in U138. (c) Extracellular Na^+^/K^+^/Cl^−^ measurements in conditioned medium pre- and post-exposure should show treatment-specific ionic shifts.

**Secondary hypothesis—AMRF-specific membrane mechanical resonance.** Clinical-trial data on AMRF in oncology motivated us to test amplitude modulation in this preclinical setting, where AMRF was found to produce different effects than unmodulated RF. We propose membrane mechanical-resonance excitation as a candidate mode of action: the fundamental mechanical-resonance frequencies $f_{M}$ of cancer-cell membranes at sub-hyperthermic temperatures fall in the audio-frequency range covered by the pink-noise AM spectrum (Ref. [3], Equation (4) and Figure 4E). For HT29, predicted $f_{M}\geq 100$ Hz lies within the AM spectrum’s support. For U138, if membrane stiffness and geometry predict $f_{M}$ in a sub-100 Hz range, this mechanism may contribute less, a pattern consistent with the cell-line-specific differences observed here. *Testable predictions:* AFM indentation of U138 at 37 °C and 41 °C to obtain Young’s modulus and estimate $f_{M}$ directly. FM1-43 or propidium–iodide uptake kinetics during AMRF as a membrane-integrity readout.

**Complementary post-transcriptional hypotheses.** For completeness, we additionally propose: (a) miRNA-mediated translational repression, which is relevant given GBM’s well-documented miRNA dysregulation (e.g., miR-21, miR-10b, miR-221/222) and is testable by small-RNA sequencing with target-protein Western blots [17,46]. (b) Extracellular-vesicle (EV) signaling, relevant given GBM’s paradigmatic EV secretion profile and testable by EV isolation (nanoparticle tracking analysis, Western for CD9/CD63/TSG101) and cargo proteomics from conditioned media [47,48,49]. And (c) post-translational metabolic rewiring (mitochondrial membrane-potential shifts, OXPHOS/glycolysis flux changes), testable by Seahorse XF extracellular-flux analysis and JC-1/TMRM mitochondrial-potential imaging within minutes to hours of AMRF exposure. These mechanisms are proposed as hypotheses for dedicated follow-up study rather than as mechanisms established by the present data.

Together, our findings indicate that amplitude modulation broadens the biological effects of RF and reveals tumor- and cell-line-specific response patterns, supporting further investigation of AMRF, alone and in combination with RT, and motivating future studies of combinations with immunotherapy or metabolic targeting.

**Limitations.** Five considerations constrain the scope of the conclusions. First, transcriptomic profiling was performed in two cell lines (SW620 and U138) rather than across all four. The cell-line-specific transcriptomic conclusions therefore apply strictly to these two models. Independent validation in HT29 and U343 by qPCR or full transcriptomic profiling is a planned extension of this work. Second, a pure-incubator control was not included, in order to maintain a consistent experimental background across all arms (Section 2.3). The 37 °C water-bath control serves as the closest matched-handling baseline. All treatment effects should be interpreted as differences relative to this handled control rather than to unperturbed incubator conditions. Third, formal analyst-level blinding to treatment condition was not implemented during phenotypic image analysis (Section 2.5). Analysis was performed with pre-set IncuCyte ZOOM parameters applied uniformly across all wells, but coded plate-map analysis with post hoc decoding would have provided a stronger methodological safeguard. Fourth, the in vitro SAR of 200–240 W/kg (Section 2.1) exceeds the 1–20 W/kg range delivered by clinical capacitive hyperthermia [3], leaving open whether the AMRF+RT synergy reported here is triggered at clinical doses. Fifth, exposures were performed in well-oxygenated suspensions and do not reproduce the hypoxic 3D microenvironment of clinical GBM, where hypoxia is itself a primary driver of radioresistance [17,18].

## 5. Conclusions

In the four cell-line models profiled, AMRF (alone and in combination with RT) was associated with biological responses exceeding those of conventional RF, HT, and RT monotherapies in most treatment-by-cell-line combinations, with AMRF+RT producing the strongest effects in three of the four lines (Figure 1, Figure 2, Figure 3 and Figure 4). Cell-line-specific exceptions identified in the Results constrain how broadly these patterns can be generalized. The findings complement clinical observations from regional HT and AMRF trials reporting improved survival without additional toxicity, and add molecular and temporal resolution to those clinical reports. They support AMRF as a candidate adjunct therapeutic strategy in the models profiled here and motivate future investigations using multi-omics approaches, multiple timepoint analyses, functional assays of the mechanisms proposed for the U138 phenotype–transcriptome divergence, and rational combination regimens.

## Figures and Tables

**Table 1:** Cell lines utilized in the experiments, along with their origin and culture media details.

Cell Line	Medium	Origin
HT29 (ATCC HTB-38) (RRID:CVCL_0320)	RPMI + 10% FBS + 1% PenStrep	Human colorectal cancer, Grade I, primary tumor
SW620 (ATCC CCL-227) (RRID:CVCL_0547)	DMEM + 10% FBS + 1% PenStrep	Human Dukes C colorectal cancer, lymph node metastasis
U343 (RRID:CVCL_S471)	DMEM + 10% FBS + 1% PenStrep	Human glioblastoma, Grade IV, primary tumor
U138 (DSMZ ACC 291) (RRID:CVCL_0020)	DMEM + 10% FBS + 1% PenStrep	Human glioblastoma, Grade IV, primary tumor

## Data Availability

The raw and processed RNA-sequencing data generated in this study are available in the NCBI Gene Expression Omnibus (GEO) under accession GSE329984. Differentially expressed gene tables for all contrasts are included as Supplementary Tables S1–S6. Further inquiries can be directed to the corresponding author.

## Supplementary Materials

The following supporting information can be downloaded at: https://www.mdpi.com/article/10.3390/cancers18101613/s1. Figure S1: SW620—RT/HT/RT+HT: Direction-specific summaries of total impacted genes per condition at 0 h and 24 h relative to control, enabling direct between-condition comparison of transcriptional impact over time. Figure S2: SW620—RF/AMRF-HT ± RT: Red and blue tracks summarize genes with increased or decreased transcription relative to control, facilitating comparison of response magnitude across RF/AMRF-HT alone and in combination with RT. Figure S3: SW620—Cross-condition comparison: Aggregated display of interventions and timepoints showing directional transcriptional changes relative to control across all SW620 conditions. Figure S4: U138—RT/HT/RT+HT: Direction-specific summaries at 0 h and 24 h relative to control, permitting direct assessment of how RT, HT, and RT+HT modulate U138 transcription over time. Figure S5: U138—RF/AMRF-HT ± RT: Intervention- and timepoint-resolved counts of up-regulated and down-regulated transcripts relative to control, enabling comparison of RF/AMRF-HT alone and in combination with RT in U138 cells. Figure S6: U138—Consolidated direct comparisons: Unified view of all interventions and timepoints in U138 with directionality and magnitude. Table S1: Differential expression results for SW620 reference therapies. Table S2: Differential expression results for U138 reference therapies. Table S3: Differential expression results for SW620 RF and AMRF plus RT. Table S4: Differential expression results for SW620 direct comparisons. Table S5: Differential expression results for U138 RF and AMRF plus RT. Table S6: Differential expression results for U138 direct comparisons.

## Abbreviations

The following abbreviations are used in this manuscript:

AMRF	Amplitude-Modulated Radiofrequency
CRC	Colorectal Cancer
ECM	Extracellular Matrix
EMF	Electromagnetic Field
GBM	Glioblastoma Multiforme
HT	Hyperthermia
KEGG	Kyoto Encyclopedia of Genes and Genomes
mEHT	Modulated Electro-Hyperthermia
RF	Radiofrequency
RT	Radiotherapy
WB	Water Bath
