# Supplementary material for: Non-Temperature-Induced Antitumor Effects of Amplitude-Modulated Radiofrequency: Molecular and Functional Synergies with Radiotherapy
Source: Cancers (Basel). 2026 May 16;18(10):1613. doi: 10.3390/cancers18101613 (PMC13204345; doi:10.3390/cancers18101613)
Supplement: Supplementary file 1 [file cancers-18-01613-s001.zip › Figure S1.pdf]

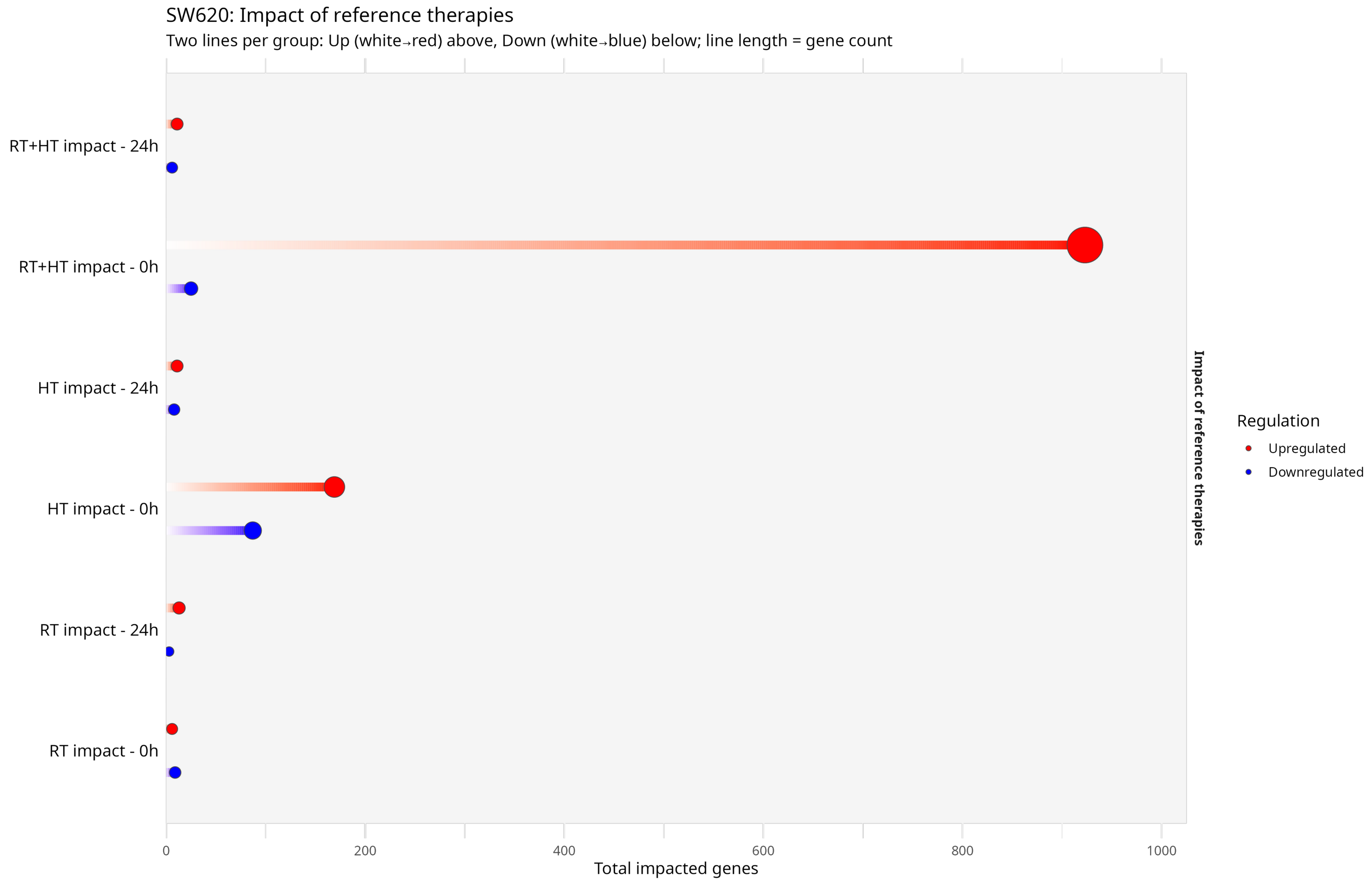

Figure S1. SW620, RT/HT/RT+HT. Comparisons at 0 h and 24 h post-treatment show the number of genes with higher (red) or lower (blue) transcription relative to the matched control for each condition. Track length and terminal marker area encode the total gene count per direction, enabling direct between-condition comparison of transcriptional impact over time. Cutoffs: $|\log_2 \mathrm{FC}| > 1$, gene adjusted $p < 0.05$, gene-set FDR < 0.05.
