# Supplementary material for: Non-Temperature-Induced Antitumor Effects of Amplitude-Modulated Radiofrequency: Molecular and Functional Synergies with Radiotherapy
Source: Cancers (Basel). 2026 May 16;18(10):1613. doi: 10.3390/cancers18101613 (PMC13204345; doi:10.3390/cancers18101613)
Supplement: Supplementary file 1 [file cancers-18-01613-s001.zip › Figure S2.pdf]

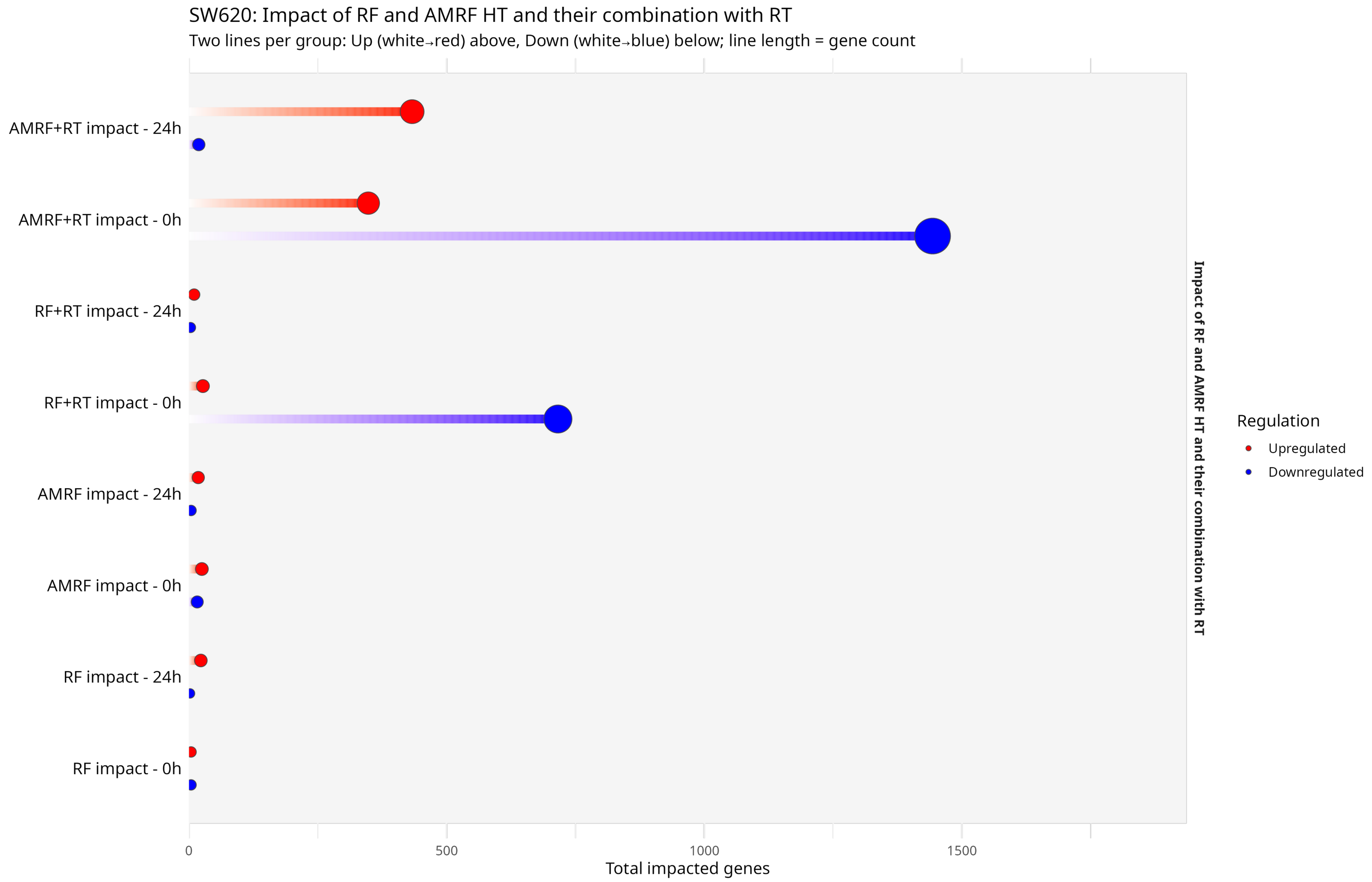

Figure S2. SW620, RF/AMRF-HT ± RT. For each intervention and timepoint, red and blue tracks summarize genes with increased or decreased transcription relative to control. The extent of each track and the size of its endpoint scale with the number of differentially transcribed genes, facilitating comparison of response magnitude across RF/AMRF-HT alone and in combination with RT. Cutoffs: $|\log_2 \mathrm{FC}| > 1$, gene adjusted $p < 0.05$, gene-set FDR < 0.05.
