# Supplementary material for: Non-Temperature-Induced Antitumor Effects of Amplitude-Modulated Radiofrequency: Molecular and Functional Synergies with Radiotherapy
Source: Cancers (Basel). 2026 May 16;18(10):1613. doi: 10.3390/cancers18101613 (PMC13204345; doi:10.3390/cancers18101613)
Supplement: Supplementary file 1 [file cancers-18-01613-s001.zip › Figure S3.pdf]

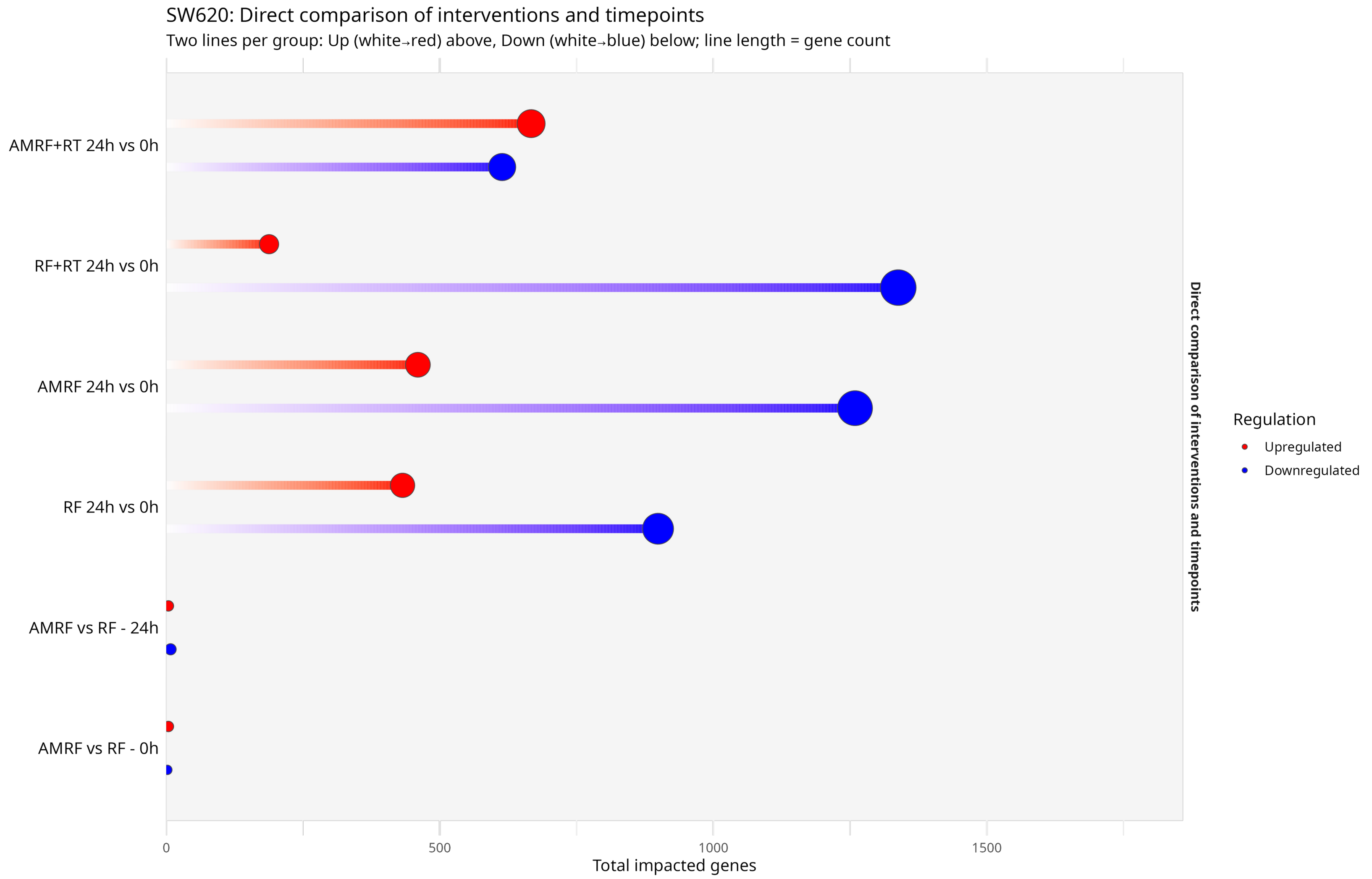

Figure S3. SW620, cross-condition comparison. Aggregated display of interventions and timepoints showing directional transcriptional changes relative to control (red for up, blue for down). Track length and endpoint size give a visual proxy for response breadth, supporting like-for-like comparison of effect sizes across all SW620 conditions. Cutoffs: $|\log_2 \mathrm{FC}| > 1$, gene adjusted $p < 0.05$, gene-set FDR < 0.05.
