# Supplementary material for: Non-Temperature-Induced Antitumor Effects of Amplitude-Modulated Radiofrequency: Molecular and Functional Synergies with Radiotherapy
Source: Cancers (Basel). 2026 May 16;18(10):1613. doi: 10.3390/cancers18101613 (PMC13204345; doi:10.3390/cancers18101613)
Supplement: Supplementary file 1 [file cancers-18-01613-s001.zip › Figure S4.pdf]

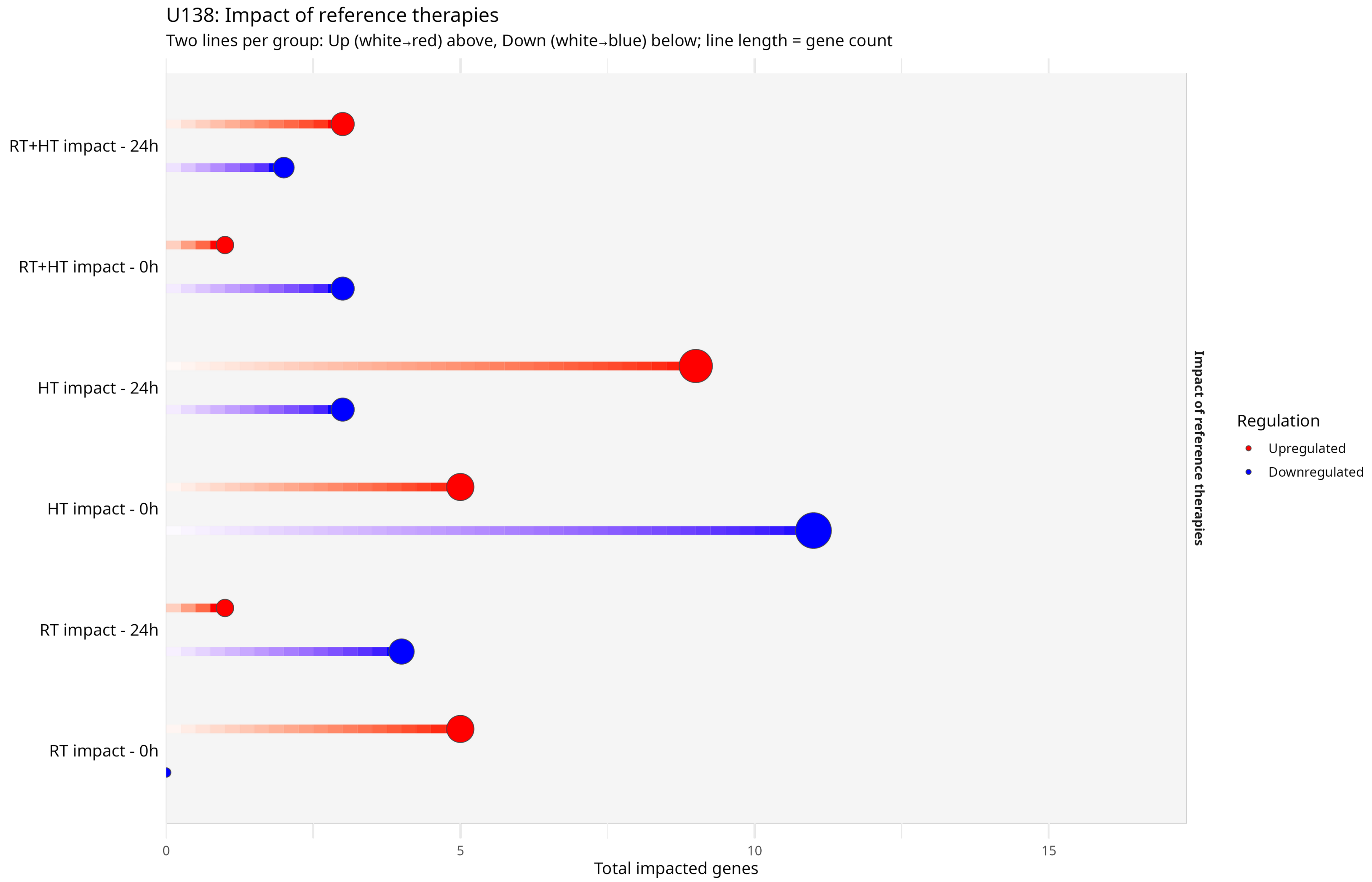

Figure S4. U138, RT/HT/RT+HT. Direction-specific summaries (up in red, down in blue) are shown at 0 h and 24 h relative to control for each reference therapy. Track length and marker area are proportional to the number of affected genes, permitting direct assessment of how RT, HT, and RT+HT modulate U138 transcription over time. Cutoffs: $|\log_2 \mathrm{FC}| > 1$, gene adjusted $p < 0.05$, gene-set FDR < 0.05.
