# Supplementary material for: Non-Temperature-Induced Antitumor Effects of Amplitude-Modulated Radiofrequency: Molecular and Functional Synergies with Radiotherapy
Source: Cancers (Basel). 2026 May 16;18(10):1613. doi: 10.3390/cancers18101613 (PMC13204345; doi:10.3390/cancers18101613)
Supplement: Supplementary file 1 [file cancers-18-01613-s001.zip › Figure S5.pdf]

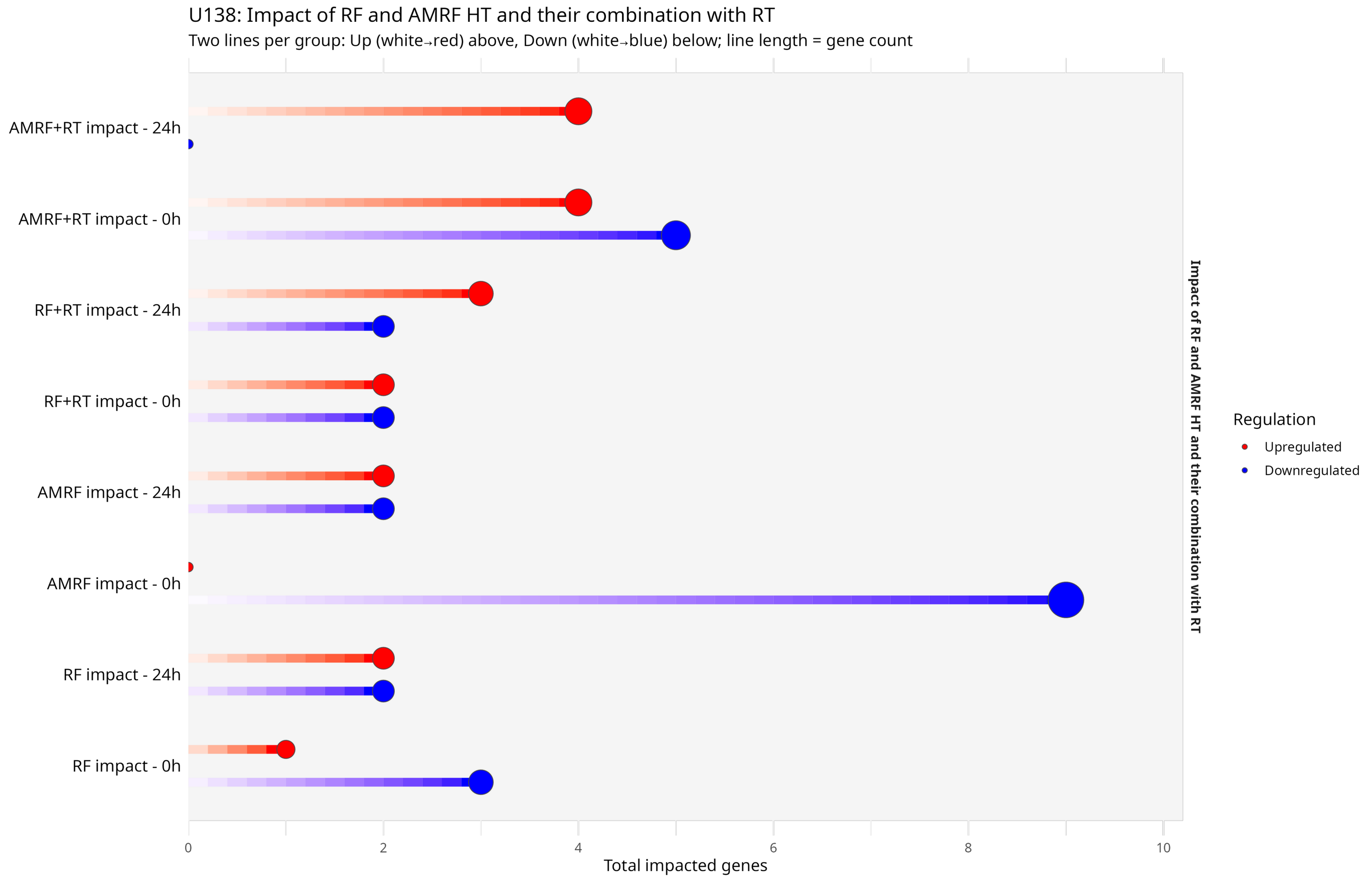

Figure S5. U138, RF/AMRF-HT ± RT. Intervention- and timepoint-resolved counts of up-regulated (red) and down-regulated (blue) transcripts relative to control. Visual encodings (track length, endpoint size) reflect the number of genes per direction, enabling comparison of RF/AMRF-HT alone and in combination with RT in U138 cells. Cutoffs: $|\log_2 \mathrm{FC}| > 1$, gene adjusted $p < 0.05$, gene-set FDR < 0.05.
