# Supplementary material for: Non-Temperature-Induced Antitumor Effects of Amplitude-Modulated Radiofrequency: Molecular and Functional Synergies with Radiotherapy
Source: Cancers (Basel). 2026 May 16;18(10):1613. doi: 10.3390/cancers18101613 (PMC13204345; doi:10.3390/cancers18101613)
Supplement: Supplementary file 1 [file cancers-18-01613-s001.zip › Figure S6.pdf]

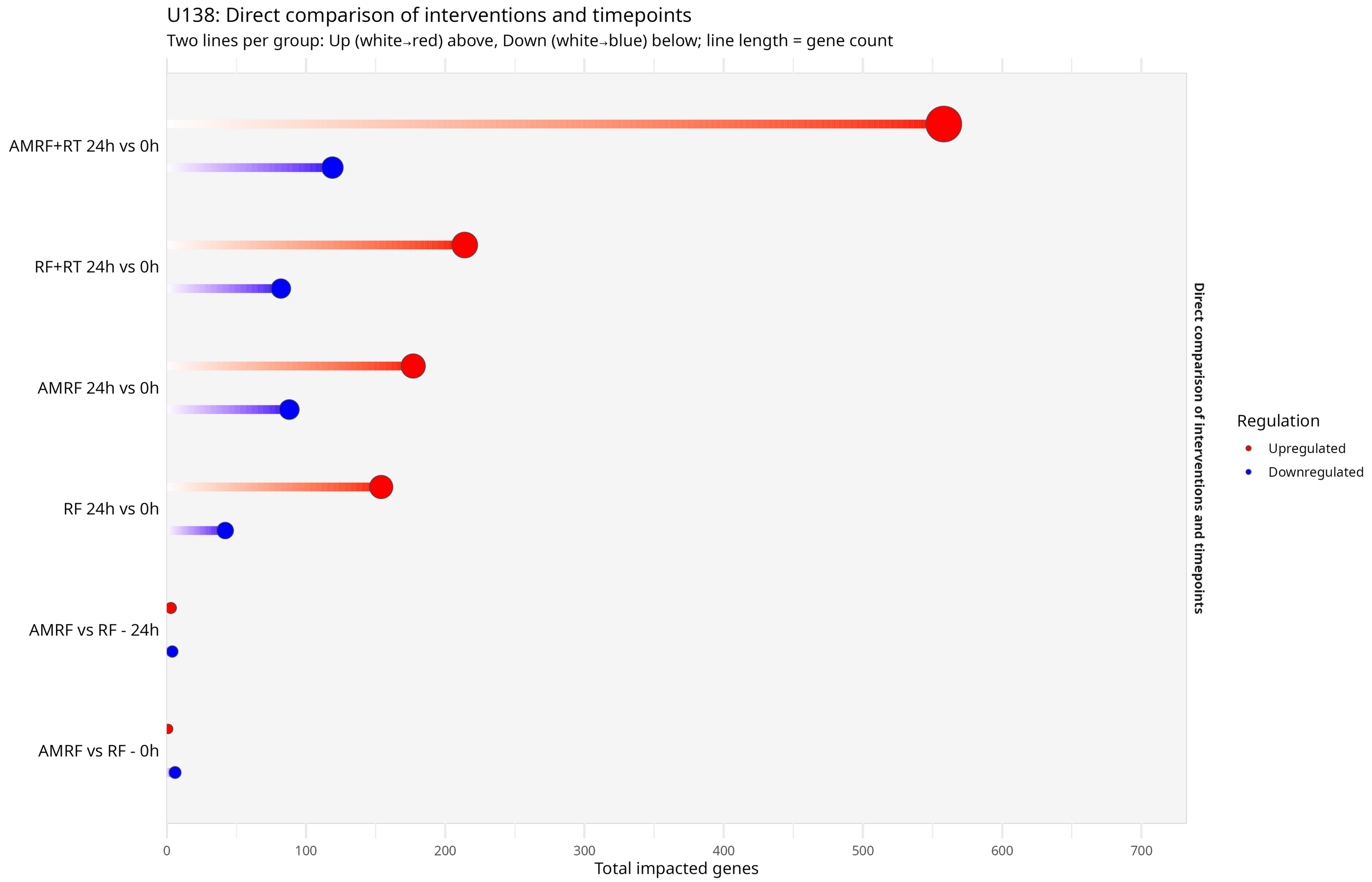

Figure S6. U138, consolidated direct comparisons. Unified view of all interventions and timepoints in U138, with direction encoded by colour (red for up, blue for down) and magnitude by track length and endpoint size. The figure gives a concise ranking of transcriptional effect sizes across conditions. Cutoffs: $|\log_2 \mathrm{FC}| > 1$, gene adjusted $p < 0.05$, gene-set FDR < 0.05.
